# Lab to Field Assessment of the Ecotoxicological Impact of Chlorpyrifos, Isoproturon, or Tebuconazole on the Diversity and Composition of the Soil Bacterial Community

**DOI:** 10.3389/fmicb.2018.01412

**Published:** 2018-06-29

**Authors:** Veronika Storck, Sofia Nikolaki, Chiara Perruchon, Camille Chabanis, Angela Sacchi, Giorgia Pertile, Céline Baguelin, Panagiotis A. Karas, Aymé Spor, Marion Devers-Lamrani, Evangelia S. Papadopoulou, Olivier Sibourg, Cedric Malandain, Marco Trevisan, Federico Ferrari, Dimitrios G. Karpouzas, George Tsiamis, Fabrice Martin-Laurent

**Affiliations:** ^1^AgroSup Dijon, Institut National de la Recherche Agronomique, Université Bourgogne Franche-Comté, Agroécologie, Dijon, France; ^2^Department of Environmental and Natural Resources Management, University of Patras, Agrinio, Greece; ^3^Laboratory of Plant and Environmental Biotechnology, Department of Biochemistry and Biotechnology, University of Thessaly, Larissa, Greece; ^4^Enoveo srl., Lyon, France; ^5^Aeiforia srl, Spinoff Università Cattolica del Sacro Cuore, Fidenza, Italy; ^6^Department of Agronomy and Environmental and Chemistry, Catholic University of the Sacred Heart, Piacenza, Italy

**Keywords:** microbial ecotoxicology, pesticide, soil, microorganisms, next-generation sequencing, DNA microarray

## Abstract

Pesticides are intentionally applied to agricultural fields for crop protection. They can harm non-target organisms such as soil microorganisms involved in important ecosystem functions with impacts at the global scale. Within the frame of the pesticide registration process, the ecotoxicological impact of pesticides on soil microorganisms is still based on carbon and nitrogen mineralization tests, despite the availability of more extensive approaches analyzing the abundance, activity or diversity of soil microorganisms. In this study, we used a high-density DNA microarray (PhyloChip) and 16S rDNA amplicon next-generation sequencing (NGS) to analyze the impact of the organophosphate insecticide chlorpyrifos (CHL), the phenyl-urea herbicide isoproturon (IPU), or the triazole fungicide tebuconazole (TCZ) on the diversity and composition of the soil bacterial community. To our knowledge, it is the first time that the combination of these approaches are applied to assess the impact of these three pesticides in a lab-to-field experimental design. The PhyloChip analysis revealed that although no significant changes in the composition of the bacterial community were observed in soil microcosms exposed to the pesticides, significant differences in detected operational taxonomic units (OTUs) were observed in the field experiment between pesticide treatments and control for all three tested pesticides after 70 days of exposure. NGS revealed that the bacterial diversity and composition varied over time. This trend was more marked in the microcosm than in the field study. Only slight but significant transient effects of CHL or TCZ were observed in the microcosm and the field study, respectively. IPU was not found to significantly modify the soil bacterial diversity or composition. Our results are in accordance with conclusions of the Environmental Food Safety Authority (EFSA), which concluded that these three pesticides may have a low risk toward soil microorganisms.

## Introduction

Soil is a limited and largely non-renewable resource providing a unique and complex habitat for a wide range of microorganisms which support key soil ecosystem functions and contribute to complex processes with impacts at the global scale (Blum, 2006; Bondeau et al., 2007; Graham et al., 2016). For instance, soil microorganisms contribute to food supply, water quality, carbon cycling, and climate regulation (Haygarth and Ritz, 2009; Hillel, 2009). In this context, the preservation of soil quality by agriculture was identified to be one of the greatest objectives of the twenty-first century (Lal, 2008; Jones et al., 2009; Lichtfouse et al., 2009; Rattan, 2015; Steffan et al., 2017). To achieve this objective, the main pressures on soil organisms were weighted by the European soil biodiversity expert group and human intensive exploitation was ranked first (Jeffery et al., 2010; Gardi et al., 2013). Pesticides are among major contributors to the pressure exerted on soil organisms. They are intentionally applied in conventional agriculture to protect crops from various pests. They can persist in soil, and from there they can be dispersed to other environmental compartments (Looser et al., 2000; Barbash, 2003; Smalling et al., 2013), and can harm non-target organisms (Pimentel, 1995; Zhang et al., 2011) including soil microorganisms as summarized by Puglisi (2012). The preservation of the soil bacterial diversity is important as it largely contributes to crucial ecosystem functions. While a well-preserved high soil bacterial diversity may ensure high rates of ecosystem processes under changing and even extreme environmental conditions, a low diversity may lead to weak ecosystem process rates under extreme environmental conditions. Thus, it is important to evaluate a potential impact of pesticides on the soil bacterial diversity and composition (Imfeld and Vuilleumier, 2012).

The evaluation of the ecotoxicological effects of pesticides on soil microorganisms remains a difficult task, as research results and their complex interpretation often differ from one study to another. Hence a more standardized approach both in terms of methods and risk assessment schemes is required. Effects of the organophosphate insecticide chlorpyrifos (CHL), the phenyl-urea herbicide isoproturon (IPU), and the triazole fungicide tebuconazole (TCZ) are particularly interesting to study as these pesticides are on the market for 53, 43 and 30 years respectively and they have been identified as common contaminants of natural water resources (Hayes and Laws, 1991; Oerke et al., 1994; Börner et al., 2009). As happens with all pesticides currently on the market, the EU regulatory assessment of the soil microbial ecotoxicity of CHL, IPU, and TCZ solely relies on tests looking at their ecotoxicological impact on carbon and nitrogen mineralization in microcosm studies. These tests, presented in the relevant regulatory documents by the Environmental Food Safety Authority (EFSA), who is in charge for the authorization of an active substance of a pesticide for all EU countries, suggest no significant impact on carbon and nitrogen mineralization in soil for CHL (EC, 2005) and effects of <25% for IPU and TCZ, which are considered as an acceptable low risk to soil microorganisms by the regulatory authorities (EFSA, 2008, 2014, 2015a). Carbon and nitrogen mineralization tests are global indicators of microbial activity which can be criticized as being relatively insensitive, hence not providing a realistic assessment of the impact of pesticides not only on microbial functions but mostly on the soil microbial diversity that should be preserved.

To date, several studies have investigated the impact of CHL, IPU, and TCZ on soil microbial mass, diversity or activity. For all three pesticies, studies on their effects were mostly performed in soil microcosms (CHL: Pozo et al., 1995; Chu et al., 2008; Fang et al., 2009; Dutta et al., 2010; Srinivasulu and Rangaswamy, 2013; IPU: Tag-El-Din, 1982; Harden et al., 1993; Kuriyal and Pandey, 1994; Perrin-Ganier et al., 2001; TCZ: Cycon et al., 2006; Bending et al., 2007; Ferreira et al., 2009; Munoz-Leoz et al., 2011; Anuradha et al., 2016; Wang et al., 2016) and seldomly at field scale (CHL: Eisenhauer et al., 2009; Gupta et al., 2013; IPU: Schuster and Schröder, 1990). Overall, most of the studies reported small to moderate (sometimes temporary) toxic effects of the three pesticides to soil microbial biomass, diversity or activity (CHL: Pozo et al., 1995; Singh et al., 2002; Chu et al., 2008; Eisenhauer et al., 2009; Fang et al., 2009; Dutta et al., 2010; Gupta et al., 2013; Srinivasulu and Rangaswamy, 2013; IPU: Tag-El-Din, 1982; Schuster and Schröder, 1990; Harden et al., 1993; Kuriyal and Pandey, 1994; Perrin-Ganier et al., 2001; TCZ: Cycon et al., 2006; Bending et al., 2007; Ferreira et al., 2009; Munoz-Leoz et al., 2011; Anuradha et al., 2016; Wang et al., 2016; TCZ: Cycon et al., 2006; Bending et al., 2007; Ferreira et al., 2009; Munoz-Leoz et al., 2011; Anuradha et al., 2016; Wang et al., 2016). It is noteworthy that several studies showed pesticide exposure to cause short-term inhibitory effects within the first month after treatment, which were recovered in subsequent months in accordance with pesticide dissipation (CHL: Pozo et al., 1995; Fang et al., 2009; TCZ: Ferreira et al., 2009; Munoz-Leoz et al., 2011).

Almost all ecotoxicological studies about pesticide effects on soil microorganisms applied classical approaches to quantify impacts on the abundance (microbial mass), structure [phospholipid fatty acid (PLFA) analysis, DNA fingerprinting approaches such as T-RFLP or A-RISA] or activity (soil respiration, soil enzymatic activity) of soil microbial communities. The latest developments of next-generation DNA sequencing (NGS) allows the extensive description of the diversity of the soil microbial community. Recent studies used NGS approaches to assess the impact of pesticides on the diversity of the soil microbial community mostly at microcosm scale level (Feld et al., 2015; Newman et al., 2016; Romdhane et al., 2016; Papadopoulou et al., 2018), while equivalent field scale studies performed under realistic exposure conditions are still lacking.

In this study, we used NGS and high-density microarrays (PhyloChip) to assess the potential ecotoxicological effects of these three pesticides on the diversity and composition of the soil bacterial community. This was done following a lab to field experimental approach, consisting of laboratory tests with higher pesticide exposure regimes (0x, 1x, 2x, and 10x the recommended agronomical pesticide dose, tier 1), and a more realistic exposure scheme at field scale (0x, 1x, 2x, and 5x the recommended dose, tier 2). To our knowledge, this is the first time that advanced molecular tools (high-density DNA microarray and 16S rDNA amplicon sequencing) were used to enable a high-resolution analysis of pesticide impacts on even less abundant members of the soil bacterial community.

## Materials and methods

### Microcosm and field study

For the soil microcosm (lab) study, soil samples from an agricultural field situated in North Italy (45°05′20.8″N 9°45′59.4″E, Google Maps), an area intensively cultivated with winter cereal, were used. The soil was characterized as loamy sand (4.2% clay, 13.5% silt, 82.2% sand) with an organic carbon content of 1.5%, a microbial biomass of 160.2 mg C/kg soil dwt and a pH of 7.5. The field had not been treated with the studied pesticides for more than 5 years, while treatment by pesticides of the same chemical group cannot be excluded although this was not documented upon personal communication with the farmer. Soil samples were collected in July 2013 from the top 20 cm of the soil profile following the procedures described by the International Standardization Organization (ISO) for collection and handling of soil samples (ISO 10381-6, 2009). The soil was manually homogenized, partially air-dried, sieved (2 mm) and stored at 4°C for ~1 week before starting the experiment. For each pesticide dose combination, three replicates were prepared that were treated with an appropriate volume of aqueous solutions of CHL, IPU or TCZ (prepared from their commercial formulations Carposan® (CHL), Quintil® (IPU), or Folicur^®;^ (TCZ), respectively) aiming pesticide doses of 1x, 2x, or 10x the recommended agronomical dose [2.0 (CHL), 1.9 (IPU), and 0.6 (TCZ) mg/kg soil dwt]. Three soil samples were treated with water instead of pesticide solutions to serve as untreated controls (0x). Altogether, 150 experimental units were prepared [(3 untreated controls + 3 pesticides × 3 concentrations × 3 replicates) × 5 time points]. The water content of all soil samples was adjusted to 40% of the water holding capacity (WHC = 46.8 wt%), which was kept constant throughout the study. Each of the 150 experimental units (microcosms) consisted of 150 g wet soil which were placed in aerated (perforated) plastic bags and incubated in the dark at 20°C. Immediately after pesticide application (0 days) and 7, 42, 56, and 100 days later, experimental units were sacrificed and stored at −80°C until processed for DNA extraction.

The field study was performed on the site from where the soil samples for the microcosm study were collected. A detailed description of the set-up of the field study is given in Papadopoulou et al., (2016). Briefly, a completely randomized block design was followed with three replicate plots of 60 m^2^ (4 × 15 m) per pesticide-dose rate combination. I.e., the field plots were randomly ordered in a checkerboard pattern. The field was seeded with a mixture of cereals (60% *Hordeum vulgare*, 25% *Triticum*, and 15% *Triticosecale*) on November 7th, 2013. Replicate plots were treated with 1x, 2x, or 5x the recommended agronomical dose of each pesticide on the 12th of November 2013, while three plots were treated with water instead to serve as untreated controls (0x). This way, 30 experimental units (field plots) were prepared (3 untreated controls + 3 pesticides × 3 concentrations × 3 replicates). Soil samples (nine sub-samples per plot bulked to a composite sample per plot and sampling day) were collected at 0, 14, 35, 70, and 105 days after treatment from the top 20 cm of the soil profile and were subsequently used for DNA extraction. Sampled soil was manually homogenized, sieved (2 mm) and kept at −80°C until DNA extraction.

### DNA extraction

DNA was extracted from 300 mg soil samples (dry weight equivalent) following the ISO 11063 procedure (2012), derived from the method described by Martin-Laurent et al. (2001). The procedure involved three principal steps: (i) microbial cell lysis by physical (bead beating) and chemical (sodium dodecyl sulfate) actions, (ii) deproteination by precipitation, and (iii) DNA precipitation, washing and purification. Soil DNA extracts were purified using PVPP-columns and then Sepharose 4B-columns according to Petric et al. (2011b).

### Microarray sample preparation and analysis

For the microcosm study, the time points 0 and 56 days after pesticide treatment were examined. For the field study, 0 and 70 days after treatment were studied. The selection of these time points was based on the dissipation and metabolic patterns of the pesticides (Papadopoulou et al., 2016) and our intention to allow ample time for recovery of the soil microbiota after potential short-term effects.

Near-full-length 16S rDNA amplification for PhyloChip analysis was carried out using universal 16S rDNA primers 27F (5′-AGAGTTTGATCM TGGCTCAG-3′)and 1492R (5′-TACGGHTACCTTGTTACGACTT-3′)(Lane, 1991). A reaction mixture of 20 μL was prepared containing the PCR buffer (Takara Mirus Bio Inc., WI), 1.5 mM MgCl_2_, 0.25 mM of each dNTP, the primers at 0.3 mM each, and 1 U Taq polymerase (Takara Mirus Bio Inc., WI). PCR reactions were performed using a PTC-200 thermocycler (MJ Research Inc., USA) with a denaturation step of 10 min at 94°C, followed by 35 cycles for library construction and 30 cycles for the PhyloChip analysis of 1 min denaturation at 94°C, 1 min primer annealing at 52°C for the library construction, eight annealing temperatures between 48 and 58°C for the PhyloChip analysis, and finally 90 s extension at 72°C. The PCR was completed by a final extension of 10 min at 72°C. The size of the PCR product was determined by agarose gel electrophoresis using appropriate size markers.

16S rDNA microarray sample preparation was performed as previously described (Tsiamis et al., 2008). Briefly, per each pool of 16S rDNA amplicons, 0.5 μg were DNase I-fragmented and biotin-labeled. An aliquot of 0.1 μg was hybridized to PhyloChip at 60 rpm at 48°C overnight. Washing, staining and scanning of the PhyloChip was done according to the manufacturer's protocol (Affymetrix, USA). PhyloTrac version 2.1 was used to remove operational taxonomic units (OTUs) present on the PhyloChip but not detected in any of the samples. An OTU was regarded as present in the sample when over 90% of its assigned probe pairs were positive (PosFrac > 0.90). Data were accessed based on the hybridization intensity score in order to calculate relative abundance and as binary presence/absence for cluster analysis. Primer6+ version 6.1.13 was used to generate principal coordinate analysis (PCoA) to examine sample groupings. Statistical significance tests between groupings were performed using Permanova as implemented within Primer6+.

### Amplicon sequencing samples preparation and data analysis

One hundred and fifty microcosm and 150 field study DNA samples [5 time points × (3 controls + 3 pesticides × 3 doses × 3 replicates)] were analyzed using an amplicon Illumina next-generation sequencing approach. In a first-step PCR, fusion primers U341F (5′-CCTACGGGRSGCAGCAG-3′) and 805R (5′-GACTACCAGGGTATCTAAT-3′) (Klindworth et al., 2012) were used to amplify the hypervariable V3-V4 region of the bacterial 16S rDNA (464 bp). Primer sequences contained corresponding overhang adapters (forward adapter: TCGTCGGCAGCGTCAGATGTGTATAAGAGACAG, reverse adapter: GTCTCGTGGGCTCGGAGATGTGTATAAGAGACAG) needed to add multiplexing index-sequences in a second-step PCR.

Concerning the samples from the microcosm study, each PCR reaction was carried out in a volume of 25 μL containing 1 μL of template DNA, 5 μL Kapa 5x High-Fidelity Hot Start Buffer, 0.3 μL of each primer (25 μM), 0.75 μL dNTPs (10 mM), High-Fidelity DNA Kapa polymerase (1 unit/μL) and 17.15 μL water. PCR reactions were performed using a PTC-200 thermocycler (MJ Research Inc., USA). Cycling conditions were 5 min at 95°C, 30 cycles at 98°C for 20 s, 60°C for 30 s, and 72°C for 45 s, followed by a final extension of 5 min at 72°C. The size of the PCR products was visually checked with agarose gel electrophoresis using appropriate size markers. The PCR products were precipitated by polyethyleneglycol (20% PEG, 2.5 M NaCl) (Hartley and Bowen, 2003) and resuspended in 15 μL water. The concentrations of the PCR products were determined with a NanodropQuawell UV-VIS spectrophotometer (Q5000). The PCR amplicons were then used as templates in a second-step PCR for addition of the multiplexing index-sequences to the overhang adapters. Amplicons were purified with AMPure XP beads and re-suspended in 30 μL water. Products were quantified using the Qubit dsDNA High-Sensitivity assay (Life Technologies), and a Bioanalyzer High-Sensitivity DNA chip (Agilent). Amplicons were pooled at equimolar concentrations and the library was quantified using a Qubit Fluorometer and dsDNA HS Assay kit (Life Technologies, CA, USA). Size-selection was done by Pippin-Prep (Sage Science), where a size range of 350–450 bp was extracted. Reaction negatives were included in the pool. Size-selected fragments were purified and quantified in the same manner as described above prior to sequencing. Sequencing was performed at IMGM SA on an Illumina MiSeq platform using a 300 bp paired-end read chemistry (Illumina).

Concerning the samples from the field study, first-step PCR amplifications were carried out in 50 μL reaction volumes containing 2.5 U of Accuzyme polymerase (Bioline), 0.2 μM of each primer, 1x AccuBuffer with 2 mM Mg^2+^, 0.5 mM of each dNTP, 0.4 μg/μL of bovine serum albumin (BSA, Sigma) (Kreader, 1996) and 2 ng of soil DNA. The thermal cycling conditions were 95°C for 5 min, followed by 35 cycles of 95°C for 15 s, 58°C for 15 s, and 72°C for 1 min, with a final extension of 72°C for 5 min. The PCR products were visualized on a 1.5% agarose gel to verify the correct size of amplicons. Amplicon sizes of randomly selected samples were further analyzed by Bioanalyzer DNA 1000 chip (Agilent Technologies). The amplicons were cleaned-up with a Nucleospin gel and PCR clean-up kit (Macherey-Nagel) following the manufacturer's instructions. Subsequently, amplicon concentrations were determined with NanoDrop 2000c (Thermo Scientific). For the addition of multiplexing index-sequences to the overhang adapters, a second-step PCR was performed using a 384 libraries Nextera XT index kit v2 (Illumina). Second-step PCR amplifications were carried out in 50 μL reaction volumes containing 1x Titanium Taq polymerase (Clontech), 1x Titanium Taq PCR buffer, 0.2 mM of each dNTP, 5 μL of each Nextera XT index primer using the TrutSeq Index Plate Fixture (Illumina). All cleaned-up amplicons from the first-step PCR were diluted to 10 ng/μL and added to the second-step PCR reaction mix at a final concentration of 1 ng/μL. Thermal cycling conditions were 96°C for 3 min, followed by 8 cycles of 95°C for 30 s, 55°C for 30 s and 72°C for 30 s, with a final extension of 72°C for 5 min. Amplicon sizes (464 bp) of randomly selected sampled were then analyzed by Bioanalyzer DNA 1000 chip (Agilent Technologies). The libraries were cleaned-up before quantification using AMPure XP beads (Beckman-Coulter Genomics) following the manufacturer's instructions. Amplicon concentrations were then fluorometrically measured with Qubit (Invitrogen) and converted to nM, assuming an average library size of 464 bp: Amplicon concentration in ng/μL × (660 g/mol × 464 bp) × 10^6^ = amplicon concentration in nM. Libraries with amplicon concentrations > 40 nM were first diluted to 40 nM and then all samples were brought to 4 nM. For pooling of the libraries, 2 μL of each sample were brought together and denatured with 0.2 N NaOH and diluted to 4 pM in hybridization buffer HT1, following the Illumina manufacturer's instructions. The pooled amplicon library was then combined with a PhiX control library (Illumina) before the Illumina MiSeq run (2 × 300 bp) was performed.

### Illumina sequencing data analysis

The raw data sequences obtained from the Illumina next-generation sequencing of the samples from the microcosm and the field study were processed using similar pipelines. Raw sequences were submitted to the NCBI under the Bioproject numbers PRJNA385243 (microcosm study) and PRJNA381453 (field study). Reads were de-multiplexed and converted to FASTQ format. For the microcosm study, Cutadapt 1.2.1 (Martin, 2011) was used to trim Illumina adapter sequences from FASTQ files. Reads were trimmed if 3 bp or more of the 3' end of a read matched the adapter sequence. Sickle version 1.200 (Joshi and Fass, 2011) was used to trim reads based on quality: Any read with a quality score of <20, or <10 bp size after trimming were discarded. Paired-end reads were assembled, trimmed by length and further corrected using PandaSeq (Masella et al., 2012). For the field study, reads were quality-controlled and assembled using PEAR (Zhang and Sun, 2014). Unassembled reads and once-assembled reads outside the expected range were discarded. All subsequent analyses were conducted in QIIME 1.9.0 (Caporaso et al., 2010a). Sequences were clustered into OTUs using USEARCH (Edgar, 2010) by open-reference OTU picking. Chimeras were detected and omitted using the program UCHIME (Edgar et al., 2011) with the QIIME-compatible version of the SILVA aligned version of the Gold database (Quast et al., 2013) for the microcosm study and with the Greengenes version of the Gold database for the field study. Taxonomy was assigned to representative sequences using the SILVA 111 release database (Quast et al., 2013) for the microcosm study and with the Greengenes 13.8 release for the field study. Representative sequences were aligned using the SILVA 111 core reference alignment using PyNAST (Caporaso et al., 2010b). In total, 4,414,311 (microcosm study) and 5,159,912 (field study) sequences in 150 samples each were analyzed using QIIME.

Several α-diversity indices, as well as indices depicting the population structure, were calculated with the QIIME pipeline (Caporaso et al., 2010a) based on the rarefied OTU table at a depth of 5,000 sequences/sample (observed species, PD whole tree, chao1 and simpson reciprocal). The indices were compared using analyses of variances (ANOVAs) in a factorial design followed by the Tukey HSD test (*p* < 0.05). Potential bacterial composition differences at phylum level were visualized using a comparative bar chart. Phyla whose total relative abundances in all samples were <0.05% were excluded from the analysis. Principal coordinate analysis (PCoA) of OTU weighted unifrac distance matrices, based on Bray-Curtis dissimilarity matrices, was performed at OTU (species) level and plotted (Hamady et al., 2010). ANOSIM analyses were performed in a factorial design to identify potentially significant differences at the community level between treatments of the same sampling day. Significantly variating OTUs were identified using the R package “pamR.” ANOVA on OTUs were followed by the Bonferroni test grouping treatments per each OTU.

## Results

### Evaluation of the impact of pesticides on the soil bacterial community with phylochip

In the microcosm study, a soil bacterial community composed of bacteria belonging to 414 families (related to the 63 bacterial phyla present on the PhyloChip) was detected. From these, six phyla were the most dominant in the microcosm study (*Proteobacteria, Firmicutes, Actinobacteria, Bacteroidetes, Chloroflexi*, and *Acidobacteria*) constituting approximately 90% of the total bacterial abundance in each sample (Supplementary Figure 1). PCoA and Permanova analyses indicated that there were no significant differences at OTU level in response to pesticide treatment, pesticide dose and time, (*p* = 0.88, *p* = 0.88, and *p* = 0.15, respectively).

**Figure 1:**
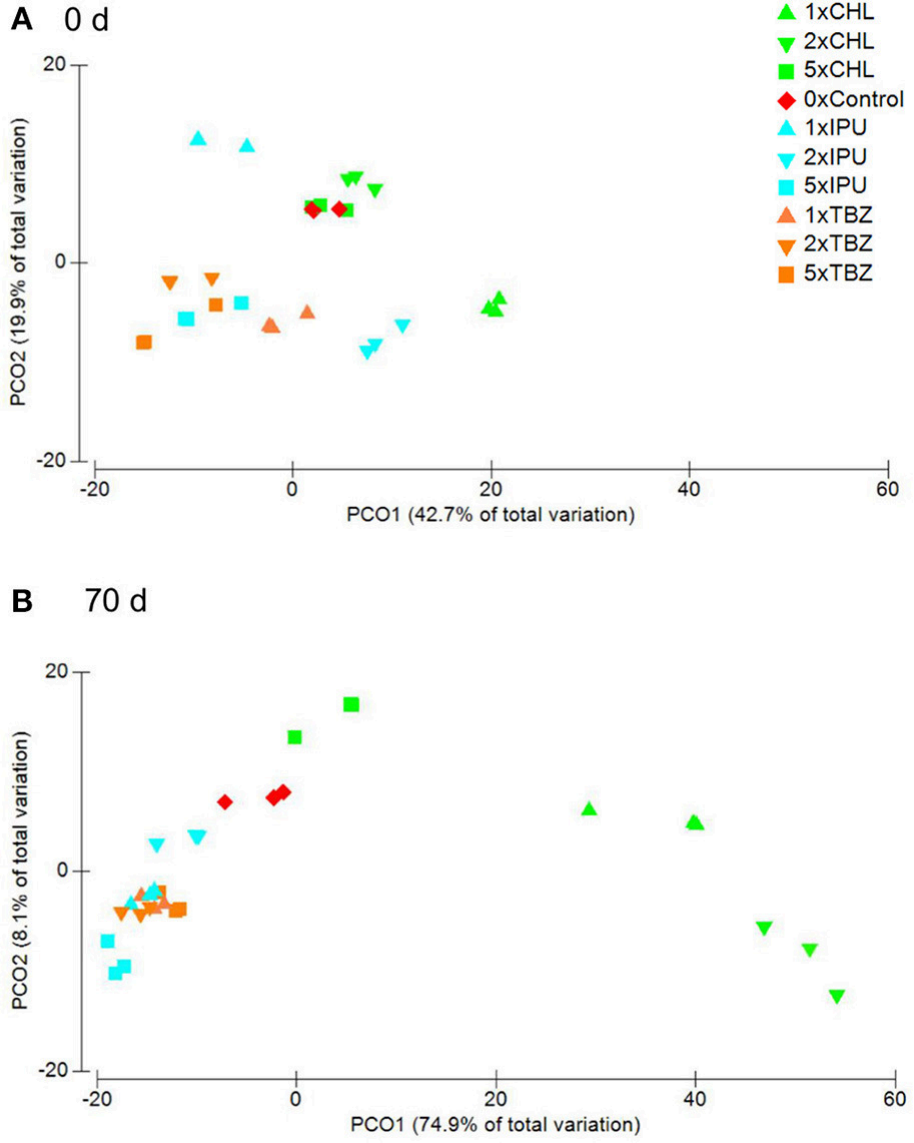
Field study—PcoA on Bray Curtis similarity matrices based on presence and absence of bacterial OTUs in controls (0x dose) and samples treated with CHL, IPU, or TCZ (1, 2x or 5x recommended agronomical doses) and collected at **(A)** 0 days or **(B)** 70 days after treatment. For each treatment values of each of the three replicates are shown. Variance is expressed in % on each axis.

In the field study, a bacterial community composed of members belonging to 402 families were detected via PhyloChip analysis. The six most dominant phyla in the field study were *Proteobacteria, Actinobacteria, Firmicutes, Cyanobacteria, Bacteroidetes*, and *Acidobacteria*, which made up around 95% of the bacterial abundance in each sample (Supplementary Figure 2). Interestingly, PCoA and Permanova analyses of the field samples at OTU level indicated a significant difference in the presence/absence of OTUs in response to the pesticide treatment (*p* = 0.013) but not for the pesticide dose (*p* = 0.458) or time 70 d after treatment (Figure 1B), while no significant differences were detected at 0 d after treatment in response to pesticide treatment and pesticide dose (*p* = 0.201, and *p* = 0.124, respectively) (Figure 1A). Interestingly, by performing pairwise tests between the pesticide treatments and the controls, significant differences were observed between the control and all three pesticides. As observed for the microcosm experiment, neither the pesticides dose nor the time had an effect on the bacterial composition when all samples of 0 d and 70 d were compared (*p* = 0.257, and *p* = 0.045, respectively).

### Evaluation of the impact of pesticides on the diversity and composition of the soil bacterial community by 16S rDNA amplicon next-generation sequencing

#### The impact of pesticides on the α-diversity of the soil bacterial community

Analysis of the α-diversity indices “observed species,” “PD whole tree,” “chao1,” and “Simpson reciprocal” indicated that the soil bacterial α-diversity was significantly affected by time in the microcosm study (Table 1), whereas time effects on the α-diversity of soil bacteria were less marked in the field study (Table 2). This observation was further confirmed by ANOSIMs of PCoAs grouping only the untreated control samples of all time points in the microcosm study (*p* = 0.001, Supplementary Figure 6, Table 3) and in the field study (*p* = 0.003, Supplementary Figure 10, Table 3), respectively.

**Table 1:** Microcosm study—Bacterial α-diversity indices “observed species, PD whole tree, chao1 and Simpson reciprocal” at various time points (0, 7, 42, 56, and 100 days after treatment) in soil exposed to each of the three studied pesticides (CHL, IPU, or TCZ) applied at different doses (0x, 1x, 2x, or 10x the recommended agronomical dose).

		**Observed species**					**PD whole tree**				
		**0 d**	**7 d**	**42 d**	**56 d**	**100 d**	**0 d**	**7 d**	**42 d**	**56 d**	**100 d**
CHL	0x	2,619^a^ ±57	2,425^ab^ ± 27	2,365^bc^ ± 11	2,191^bc^ ± 31	2,188^c^ ± 43	227^a^ ± 5	211^ab^ ± 2	212^ab^c ± 2	191^bc^ ± 2	198^c^ ± 5
	1x	2,577^a^ ± 77	2,327^ab^ ± 11	2,268^bc^ ± 40	2,301^bc^ ± 47	2,139^c^ ± 21	228^a^ ± 2	214^ab^ ± 8	210^abc^ ± 2	206^bc^ ± 5	188^c^ ± 2
	2x	2,581^a^ ± 60	2,447^ab^ ± 24	2,282^bc^ ± 19	2,283^bc^ ± 34	2,131^c^ ± 29	223^a^ ± 7	213^ab^ ±5	202^abc^ ± 2	205^bc^ ± 2	191^c^ ± 5
	10x	2,539^a^ ± 91	2,380^ab^ ± 20	2,360^bc^ ± 35	2,187^bc^ ± 37	2,157^c^ ± 17	224^a^ ± 2	214^ab^ ± 9	204^ab^c ± 5	196^bc^ ± 5	193^c^ ± 2
IPU	0x	2,619^a^ ± 57	2,425^ab^ ± 27	2,365^bc^ ± 28	2,191^bc^ ± 31	2,188^c^ ± 43	227^a^ ± 5	211^ab^ ± 2	212^ab^ ± 2	191^c^ ± 2	198^bc^ ± 5
	1x	2,663^a^ ± 41	2,398^ab^ ± 92	2,386^bc^ ± 35	2,251^bc^ ± 15	2,200^c^ ± 60	230^a^ ± 7	212^ab^ ± 12	216^ab^ ± 4	198^c^ ± 1	195^bc^ ± 6
	2x	2,406^a^ ± 241	2,322^ab^ ± 67	2,272^bc^ ± 30	2,237^bc^ ± 24	2,166^c^ ± 13	205^a^ ± 23	204^ab^ ± 8	204^ab^ ± 3	192^c^ ± 2	193^bc^ ± 2
	10x	2,584^a^ ± 115	2,448^ab^ ± 23	2,353^bc^ ± 25	2,184^bc^ ± 35	2,148^c^ ± 18	223^a^ ± 9	217^ab^ ± 2	212^ab^ ± 3	191^c^ ± 3	198^bc^ ± 2
TCZ	0x	2,619^a^ ± 57	2,425^ab^ ± 27	2,365^bc^ ± 28	2,191^c^ ± 31	2,188^c^ ± 43	227^a^ ± 5	211^ab^ ± 2	212^bc^ ± 2	191^c^ ± 2	198^c^ ± 5
	1x	2,630^a^ ± 23	2,444^ab^ ± 77	2,323^bc^ ± 21	2,100^c^ ± 26	2,134^c^ ± 32	216^a^ ± 11	202^ab^ ± 4	202^bc^ ± 5	185^c^ ± 4	191^c^ ± 3
	2x	2,575^a^ ± 77	2,403^ab^ ± 35	2,249^bc^ ± 24	2,226^c^ ± 25	2,172^c^ ± 26	222^a^ ± 6	216^ab^ ± 2	202^bc^ ± 2	191^c^ ± 2	196^c^ ± 1
	10x	2,624^a^ ± 15	2,412^ab^ ± 89	2,305^bc^ ± 60	2,279^c^ ± 38	2,118c ± 50	219^a^ ± 5	210^ab^ ± 4	210^bc^ ± 3	201^c^ ± 5	187^c^ ± 6
		**Chao 1**					**Simpson reciprocal**				
CHL	0x	6,727^a^ ± 483	6,516^a^ ± 173	6,310^a^ ± 173	5,987^a^ ± 139	6,319^a^ ± 416	774^a^ ± 13	421^b^ ± 16	440^b^ ± 14	259^b^ ± 14	299^b^ ± 28
	1x	6,590^a^ ± 528	5,910^a^ ± 189	6,206^a^ ± 337	7,053^a^ ± 486	5,847^a^ ± 144	731^a^ ± 34	430^b^ ± 31	329^b^ ± 23	382^b^ ± 22	351^b^ ± 12
	2x	6,241^a^ ± 414	6,559^a^ ± 201	5,873^a^ ± 188	6,793^a^ ± 183	5,761^a^ ± 209	758^a^ ± 37	537^b^ ± 12	420^b^ ± 9	427^b^ ± 18	357^b^ ± 16
	10x	6,401^a^ ± 249	6,039^a^ ± 266	6,673^a^ ± 273	5,838^a^ ± 408	5,771^a^ ± 165	589^a^ ± 147	475^b^ ± 30	354^b^ ± 19	367^b^ ± 9	336^b^ ± 11
IPU	0x	6,727^a^ ± 483	6,516^a^ ± 173	6,310^a^ ± 173	5,987^a^ ± 139	6,319^a^ ± 416	774^a^ ± 13	421^b^ ± 16	440^b^ ± 14	259^b^ ± 14	299^b^ ± 28
	1x	6,909^a^ ± 359	6,317^a^ ± 608	6,570^a^ ± 355	6,213^a^ ± 161	5,974^a^ ± 313	817^a^ ± 29	439b ± 16	411^b^ ± 35	349^b^ ± 74	346^b^ ± 35
	2x	5,830^a^ ± 1,346	5,745^a^ ± 513	5,703^a^ ± 360	6,174^a^ ± 207	5,714^a^ ± 147	680^a^ ± 21	423^b^ ± 11	434^b^ ± 13	379^b^ ± 19	380^b^ ± 16
	10x	6,606^a^ ± 753	5,745^a^ ± 513	6,458^a^ ± 251	5,938^a^ ± 210	6,129^a^ ± 197	701^a^ ± 53	457^b^ ± 16	452^b^ ± 13	335^b^ ± 14	337^b^ ± 10
TCZ	0x	6,727^a^ ± 483	6,516^ab^ ± 173	6,310^ab^ ± 173	5,987^ab^ ± 139	6,319^b^ ± 416	774^a^ ± 13	421^b^ ± 16	440^b^ ± 14	259^b^ ± 14	299^b^ ± 28
	1x	6,995^a^ ± 179	6,257^ab^ ± 275	6,029^ab^ ± 115	5,448^a^b ± 152	6,082^b^ ± 154	787^a^ ± 29	540^b^ ± 93	425^b^ ± 29	309^b^ ± 8	345^b^ ± 12
	2x	6,535^a^ ± 610	6,317^ab^ ± 234	5,681^ab^ ± 269	6,289^ab^ ± 198	5,898^b^ ± 150	680^a^ ± 93	447^b^ ± 22	377^b^ ± 10	345^b^ ± 14	357^b^ ± 11
	10x	6,956^a^ ± 176	6,221^ab^ ± 610	6,302^ab^ ± 386	6,815^ab^ ± 330	5,721^b^ ± 273	742^a^ ± 29	507^b^ ± 22	407^b^ ± 23	386^b^ ± 15	343^b^ ± 18

*For each diversity index, ANOVAs followed by the Tukey HSD test, (p < 0.05) were done by grouping (for each tested pesticide) the five sampling time points and the four pesticide doses. Significant differences are indicated by different letters*.

**Table 2:** Field study—Bacterial α-diversity indices “observed species, PD whole tree, chao1 and Simpson reciprocal” at various time points (0, 14, 35, 70, and 105 days after treatment) in soil exposed to each of the three studied pesticides (CHL, IPU, or TCZ) applied at different doses (0x, 1x, 2x, or 5x the recommended agronomical dose).

		**Observed species**					**PD whole tree**				
		**0 d**	**14 d**	**35 d**	**70 d**	**105 d**	**0 d**	**14 d**	**35 d**	**70 d**	**105 d**
CHL	0x	1,832^abc^ ± 82	2,224^a^ ± 273	1,997^abc^ ± 109	1,735^abc^ ± 151	1,862^abc^ ± 81	137^abc^ ± 5	180^a^ ± 24	154^abc^ ± 8	133^bc^ ± 10	143^abc^ ± 3
	1x	1,655^bc^ ± 82	1,924^abc^ ± 55	2,123^ab^ ± 136	1,913^abc^ ± 68	1,926^abc^ ± 59	128^bc^ ± 6	146^abc^ ± 2	160^ab^ ± 11	148^abc^ ± 5	148^abc^ ± 5
	2x	1,561^c^ ± 95	1,875^abc^ ± 69	1,866^abc^ ± 91	1,789^abc^ ± 38	1,851^abc^ ± 20	121^c^ ± 6	144^abc^ ± 4	139^abc^ ± 3	138^abc^ ± 5	140^abc^ ± 1
	10x	1,872^abc^ ± 21	1,983^abc^ ± 93	2,067^abc^ ± 27	1,662^bc^ ± 83	1,711^abc^ ± 61	141*abc* ± 2	150^abc^ ± 7	155^abc^ ± 3	131^bc^ ± 5	132^bc^ ± 5
IPU	0x	1,823^ab^ ± 82	2,224^a^ ± 273	1,997^abc^ ± 109	1,735^ab^ ± 151	1,862^ab^ ± 81	137^abc^ ± 5	180^a^ ± 24	154^ab^ ± 8	133^abc^ ± 10	143^ab^ ± 3
	1x	1,564^b^ ± 82	18,939^ab^ ± 19	1,974^a^ ± 104	1,888^ab^ ± 70	1,843^ab^ ± 81	122^b^ ± 5	141^ab^ ± 2	150^ab^ ± 7	148^ab^ ± 4	141^ab^ ± 3
	2x	1,916^ab^ ± 11	1,755^ab^ ± 136	1,785^ab^ ± 137	1,794^ab^ ± 104	1,907^ab^ ± 11	147^ab^ ± 2	132^b^ ± 9	139^abc^ ± 8	142^ab^ ± 6	144^ab^ ± 1
	10x	1,890^ab^ ± 117	1,797^ab^ ± 110	1,828^ab^ ± 84	1,935^ab^ ± 75	1,890^ab^ ± 67	143^ab^ ± 8	135^ab^ ± 7	142^ab^ ± 4	150^ab^ ± 5	142^ab^ ± 3
TCZ	0x	1,823^a^ ± 82	2,224^a^ ± 273	1,997^abc^ ± 109	1,735^a^ ± 151	1,862^a^ ± 81	137^abc^ ± 5	180^a^ ± 24	154^ab^ ± 8	133^b^ ± 10	143^ab^ ± 3
	1x	1,871^a^ ± 71	1,931^a^ ± 107	1,840^a^ ± 98	1,870^a^ ± 41	1,894^a^ ± 18	145^ab^ ± 5	144^ab^ ± 7	144^ab^ ± 7	143^ab^ ± 3	142^ab^ ± 1
	2x	2, 044^a^ ± 26	1,963^a^ ± 41	1,935^a^ ± 124	1,885^a^ ± 49	1,889^a^ ± 52	156^ab^ ± 1	148^ab^ ± 2	151^ab^ ± 9	145^ab^ ± 2	143^ab^ ± 2
	10x	1,816^a^ ± 9	1,869^a^ ± 53	1,924 ± 84	1,807^a^ ± 67	1,780^a^ ± 12	135^b^ ± 2	142^ab^ ± 3	147^ab^ ± 6	141^ab^ ± 3	136^b^ ± 3
		**Chao 1**					**Simpson reciprocal**				
CHL	0x	2, 887^ab^ ± 193	4, 227^a^ ± 865	3, 374^ab^ ± 481	2, 787^ab^ ± 386	2, 907^ab^ ± 132	687^abc^ ± 52	774^a^ ± 60	731^abc^ ± 44	489^c^ ± 51	668^abc^ ± 49
	1x	2, 475^b^ ± 161	3, 180^ab^ ± 122	3, 878^ab^ ± 586	3, 257^ab^ ± 279	3, 026^ab^ ± 134	568^abc^ ± 31	680^abc^ ± 28	670^abc^ ± 55	506^bc^ ± 46	754^ab^ ± 45
	2x	2, 356^b^ ± 143	2, 941^ab^ ± 196	2, 912^ab^ ± 257	2, 837^ab^ ± 86	2, 929^ab^ ± 62	547^abc^ ± 62	606^abc^ ± 34	611^abc^ ± 23	516^abc^ ± 23	661^abc^ ± 47
	10x	3, 203^ab^ ± 146	3, 448^ab^ ± 306	3, 646^ab^ ± 78	2, 626^ab^ ± 163	2, 680^ab^ ± 115	542^abc^ ± 47	524^abc^ ± 7	650^abc^ ± 46	549^abc^ ± 52	589^abc^ ± 15
IPU	0x	2, 887^ab^ ± 193	4, 227^a^ ± 865	3, 374^ab^ ± 481	2, 787^ab^ ± 386	2, 907^ab^ ± 132	687^ab^ ± 52	774^a^ ± 60	731^ab^ ± 44	489^ab^ ± 51	668^ab^ ± 49
	1x	2, 323^b^ ± 174	2, 976^ab^ ± 76	2, 405^ab^ ± 382	3, 056^ab^ ± 228	3, 033^ab^ ± 99	542^ab^ ± 43	640^ab^ ± 29	644^ab^ ± 50	592^ab^ ± 53	645^ab^ ± 41
	2x	3, 145^ab^ ± 161	2, 854^ab^ ± 346	2, 818^ab^ ± 345	2, 762^ab^ ± 222	3, 180^ab^ ± 114	607^ab^ ± 56	529^ab^ ± 50	636^ab^ ± 33	656^ab^ ± 26	615^ab^ ± 17
	10x	3, 088^ab^ ± 291	2, 934^ab^ ± 266	2, 924^ab^ ± 214	3, 175^ab^ ± 172	3, 170^ab^ ± 219	672^ab^ ± 56	585^ab^ ± 90	630^ab^ ± 40	669^ab^ ± 45	624^ab^ ± 9
TCZ	0x	2, 887^ab^ ± 193	4, 227^a^ ± 865	3, 374^a^ ± 481	2, 787^a^ ± 386	2, 907^a^ ± 132	687^a^ ± 52	774^a^ ± 60	731^a^ ± 44	489^a^ ± 51	668^a^ ± 49
	1x	3, 027^ab^ ± 129	3, 133^a^ ± 301	3, 069^a^ ± 333	2, 979^a^ ± 112	3, 054^a^ ± 90	700^a^ ± 85	628^a^ ± 60	591^a^ ± 21	621^a^ ± 45	633^a^ ± 35
	2x	3, 482^ab^ ± 95	3, 350^a^ ± 200	3, 170^a^ ± 382	3, 074^a^ ± 164	3, 088^a^ ± 128	722^a^ ± 22	667^a^ ± 32	691^a^ ± 28	697^a^ ± 48	638^a^ ± 37
	10x	3, 000^ab^ ± 36	2, 975^a^ ± 115	3, 175^a^ ± 237	2, 780^a^ ± 190	2, 802a ± 38	601^a^ ± 63	671^a^ ± 42	671^a^ ± 42	611^a^ ± 17	580^a^ ± 25

*For each diversity index, ANOVAs followed by the Tukey HSD test, (p < 0.05) were done by grouping (for each tested pesticide) the five sampling time points and the four pesticide doses. Significant differences are indicated by different letters*.

**Table 3:** Microcosm and field study—ANOSIM of the PCoA ordinations of OTU weighted unifrac distance matrices for untreated (control) and treated [CHL, IPU or TCZ at 1x, 2x, or 10x (microcosms) or 1x, 2x, or 5x (field) doses] soil samples at different time points [0, 7, 42, 56, and 100 days (microcosms) or 0, 14, 35, 70 and 105 days (field)].

		**Microcosm study**		**Field study**	
	**Days**	***p-*value**	**Statistic test**	***p*-value**	**Statistic test**
CHL	0	0.738	−0.056	0.116	0.173
	7	0.250	0.250	0.100	0.191
	42	0.111	0.127	0.707	−0.099
	56	0.001	0.593	0.106	0.123
	100	0.117	0.191	0.855	−0.151
IPU	0	0.453	−0.077	0.031	0.312
	7	0.548	−0.022	0.077	0.191
	42	0.325	0.049	0.126	0.194
	56	0.373	0.022	0.015	0.355
	100	0.099	0.151	0.025	0.378
TCZ	0	0.511	−0.003	0.979	−0.179
	7	0.030	0.296	0.040	0.216
	42	0.046	0.228	0.001	0.676
	56	0.183	0.096	0.052	0.201
	100	0.028	0.250	0.110	0.244
Control	All	0.001	0.548	0.003	0.418

*Significant differences between groups are indicated by p < 0.01*.

In contrast, regardless of the applied pesticide or dose, no significant differences in any of the diversity indices between control (0x) and pesticide-treated (1x, 2x, 10x) soil microcosms were recorded at each time point at both experimental scales (microcosm and field study), indicating that none of the three pesticides significantly modified the α-diversity of the soil bacterial community (Tables 1, 2).

#### The impact of pesticides on the β-diversity of the soil bacterial community

**Figure 2:**
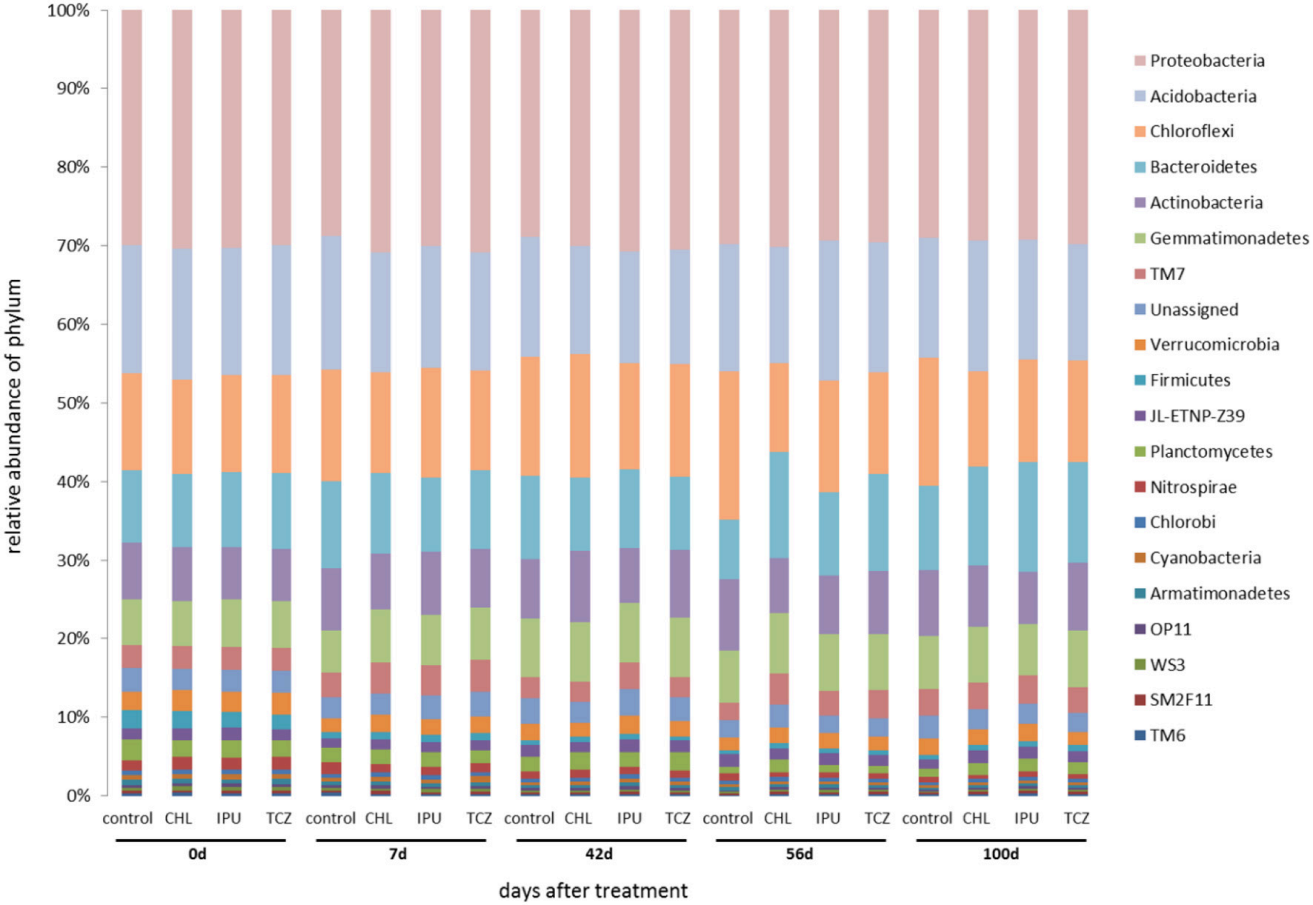
Microcosm study—The relative abundance (%) of bacterial phyla in the untreated (control) and pesticide-treated (CHL, IPU, or TCZ at 10x dose) soil samples at different time points (0, 7, 42, 56, and 100 days after treatment). 16S rDNA sequences that could not be assigned to a phylum were grouped as “unassigned”.

**Figure 3:**
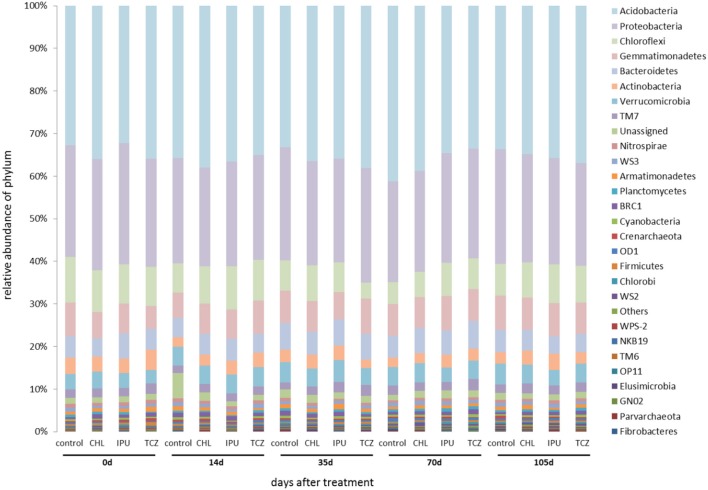
Field study—The relative abundance (%) of bacterial phyla in the untreated (control) and pesticide-treated (CHL, IPU, or TCZ at 5x dose) soil samples at different time points (0, 14, 35, 70, and 105 days after treatment). Bacterial phyla whose abundance sum of all samples was <0.05% were grouped as “others.” 16S rDNA sequences that could not be assigned to a phylum were grouped as “unassigned”.

Based on the amplicon NGS analysis, *Proteobacteria, Acidobacteria, Chloroflexi, Bacteroidetes, Actinobacteria*, and *Gemmatimonadetes* constituted the six most abundant bacterial phyla in the soil samples from the microcosm and the field experiment representing together up to 85% of the abundance of all bacterial phyla. Further analysis showed that neither the applied pesticide and dose nor the time significantly affected the abundance of the most dominant bacterial phyla in the soil (Figures 2, 3).

A more extensive analysis of the 16S rDNA amplicon sequences was performed by ANOSIMs (Table 3) on PCoAs (Supplementary Figures 3–10) using OTU weighted unifrac distance matrices. This analysis verified the lack of highly significant pesticide-induced differences in the composition of the soil bacterial community. However, at certain time points in the microcosm and the field study, transient pesticide-driven effects were identified. In the microcosm study, a significantly modified (*p* < 0.01) bacterial community was observed at 56 days in the CHL-treated samples (all three doses grouped together) compared to the corresponding untreated controls (Table 3, Supplementary Figure 3). Similarly, in the field study, TCZ induced significant transient changes (p < 0.01) in the composition of the soil bacterial community at 35 days (Table 3, Supplementary Figure 9). No significant changes in the soil bacterial β-diversity were observed in response to IPU-treatment in both microcosm and field experiments.

Analysis of the detected OTUs by the R package “pamR” was employed to identify the OTUs driving the significant changes detected by ANOSIM in the β-diversity of bacteria in the CHL- or TCZ-treated samples collected at 35 or 56 days from the field or microcosm study, respectively. In the microcosm study, eight OTUs were responsible for the differences between CHL-treated samples and untreated samples at 56 days: unknown species of the (i) genus *Aeromicrobium*, (ii) class *Acidobacteria*, (iii) genus *Flexibacter*, (iv)order *Chloroacidobacterium*, (v) order *Sphingobacteriales*, and three unknown species of the phylum *Chloroflexi* (Supplementary Figure 11). In the field study, two OTUs were found to be responsible for differences between TCZ-treated and control samples at 35 days: unknown species of the (i) class *Acidobacteria* and (ii) the order *Acidimicrobiales* (Supplementary Figure 12).

## Discussion

The study evaluated at both lab and field scale the ecotoxicological impact of three commonly used pesticides, CHL, IPU, or TCZ, on the soil bacterial diversity initially using PhyloChip, a high density phylogenetic microarray, and then further by Illumina next-generation sequencing of 16S rDNA amplicons. Both approaches revealed that *Proteobacteria, Acidobacteria, Bacteroidetes*, and *Actinobacteria* dominated the total bacterial community in both the microcosm and the field study. This is in accordance with various previous studies which also reported the dominance of these phyla in a range of soils (Janssen, 2006; Acosta-Martinez et al., 2008; Wessen et al., 2010; Petric et al., 2011a; Merlin et al., 2015; Romdhane et al., 2016). The abundance of these phyla remained relatively constant throughout the microcosm and the field study.

### Time effects more distinctive in the microcosm study than in the field study

Diversity indices in the control samples (not exposed to pesticides) significantly decreased with time in the microcosm study. This decrease trend in the bacterial diversity was not seen in the field study, where only a few time points of some diversity indices were affected by time without showing a clear pattern of decrease. This is in accordance with one of the limitations of long-term soil microcosm experiments causing changes in microbial abundance, composition, diversity and activity due to the lack of nutrient inputs (Edwards et al., 1997). In our study, the duration of the microcosm experiment was extended to 100 days in order to accommodate the long persistence of CHL, IPU and TCZ (10x dose rate) in the given soil with DT_90s_ of 96, 85 and 316 days, respectively (Papadopoulou et al., 2016).

The changes in α-diversity observed in the soil samples collected from the untreated plots in the field experiment could not be assigned to shortage of nutrients but most likely to the climatic conditions which were characterized by long cold periods (mean daily temperature <4°C between 15 and 70 days) and precipitation events at days 3 to 11 which might be responsible for the higher α-diversity values observed at 14 days. PCoA and ANOSIM analyses of the data from amplicon sequencing data at OTU level confirmed the significant effect of time on bacterial diversity in both experimental scales, with a more distinctive time effect on the β-diversity in the microcosm study (p = 0.001) than in the field study (*p* = 0.003).

### Pesticide effects on the soil bacterial diversity

Although no systematic changes in the diversity and composition of the soil bacterial community were observed in response to CHL exposure, amplicon sequencing analysis identified a slight but significant change in the β-diversity of soil bacteria at 56 days in the microcosm experiment. Interestingly, this coincided with the formation of the maximum levels of 3,5,6-trichloropyridinol (TCP), the main hydrolysis product of CHL, in the soil which received the 10x dose (Papadopoulou et al., 2016). In contrast, in the field experiment, where CHL rapidly dissipated and high TCP concentrations were not observed (probably due to TCP leaching toward groundwater out of the examined soil layer), both the α- and β-diversity was not significantly altered when examined by NGS (although PhyloChip analysis proposed a slight but significant CHL effect at 70 days in the field study). One could therefore hypothesize that (i) changes in the bacterial diversity recorded at 56 days in the microcosm experiment in response to CHL exposure may not only be due to CHL (that had partially dissipated by that time) but could be also assigned to the formation of TCP and that (ii) resilience resulting in the recovery of the bacterial diversity by the end of the study may be attributed to the rapid dissipation of TCP and the gradual dissipation of CHL. This is in accordance with a number of studies reporting the toxicity of TCP to (micro)organisms (Feng et al., 1997; Cáceres et al., 2007). Although the review report of the European Commission (EC, 2005) and other studies (Singh et al., 2002) concluded that CHL does not impose unacceptable effects on soil microorganisms, our study reports a slight but significant ecotoxicological effect of CHL on the soil bacterial β-diversity only in the microcosm experiment (when analyzed by NGS), probably because CHL and TCP cannot dissipate out of the tested soil layer by transport processes (leaching, run-off, volatilization), as it can be the case in field experiments where CHL was not found to modify the bacterial composition when analyzed by NGS. The effect of CHL was only transient and the soil bacterial β-diversity recovered. The biological importance (as compared to other environmental stresses such as extreme weather conditions) and the consequences of this transient modification of the diversity in response to CHL exposure on the functions supported by the bacterial community remain unknown. Our findings are in the line with previous studies which also observed an impact of CHL on the diversity of soil bacteria using molecular fingerprinting (Gilani et al., 2010; Gupta et al., 2013) or PLFAs (Pozo et al., 1995). However, most of the studies did not investigate the parallel formation of pesticide transformation products which might have a secondary effect on soil microorganisms, and also relied on methods which do not have the discrimination capacity of PhyloChip and mostly amplicon sequencing.

IPU exposure did not affect the soil bacterial diversity, as determined by amplicon sequencing, in both microcosms and field experiments. Similar results were obtained by the PhyloChip with the only exception of the slight but significant effect of IPU on the presence/absence of OTUs at 70 d in the field experiment. Overall, our findings are in agreement with the conclusion of EFSA (2015b) “that IPU shows no unacceptable effect on carbon and nitrogen mineralization” and with other studies reporting small to moderate (sometimes temporary) effects of IPU on soil microorganisms (mainly based on decreasing microbial activity and biomass) (Tag-El-Din, 1982; Schuster and Schröder, 1990; Harden et al., 1993; Kuriyal and Pandey, 1994).

In the field experiment, TCZ exposure induced slight but significant changes in the β-diversity of the bacterial community after 35 days (NGS outcome) and 70 days (PhyloChip outcome) of exposure in the field study. TCZ was rapidly dissipated in the field experiment (60% dissipation after 35 days) (Papadopoulou et al., 2016). In addition, an important number and variety of TCZ transformation products, including triazole dead-end transformation products possibly interacting with the hormone regulation network of non-target organisms (Shalini et al., 2011; Rieke et al., 2014), were detected but not quantified (due to the absence of appropriate reference standards) in the samples of the field experiment (treated with the 5x agronomical dose) (Storck et al., 2016). Therefore, one could propose that observed changes in the bacterial composition are not only due to exposure to TCZ but also to its transformation products. Nevertheless, although the late resilience of the bacterial composition was not addressed by PhyloChip, one could observe by NGS results that the bacterial β-diversity was resilient suggesting the recovery of the bacterial community. Although the evaluations of EFSA (2008, 2014) for TCZ concluded that the fungicide does not entail an unacceptable risk for soil microorganisms (based on carbon and nitrogen mineralization tests), our study reports slight but significant and transient effect of TCZ on the composition of the bacterial community in the field experiment. This is in agreement with previous studies reporting TCZ effects on soil microorganisms, evaluated by analysis of soil enzyme activities (Anuradha et al., 2016), microbial activity and biomass (Cycon et al., 2006; Bending et al., 2007; Munoz-Leoz et al., 2011; Wang et al., 2016), or DGGE fingerprinting (Ferreira et al., 2009).

Our study showed that small and transient changes in the diversity of soil bacteria can be detected for two (CHL and TCZ) of the three tested pesticides by 16S rDNA amplicon NGS, while the use of PhyloChip indicated significant changes in response to all three tested pesticides only for the field study. The main advantage of the PhyloChip lies on its great sensitivity for rare species (Nikolaki and Tsiamis, 2013). Indeed, one could estimate that 500 ng of 16S rDNA PCR product comprised more than 600 billion sequences enabling the detection of not only dominant but also of rare amplicons (Katsaveli et al., 2012; Nikolaki and Tsiamis, 2013). On the contrary, the main disadvantages of PhyloChip are that only known phylotypes can be detected and that signals obtained for dominant phylotypes can be saturated leading to underestimation of their abundance.

Contrariwise to Romdhane et al. (2016) reporting big shifts in the bacterial abundance and diversity exposed to leptospermone, a triketone herbicide of natural origin, only slight effects were observed in our study. This may be due to the fact that, in contrary to a natural triketone for which the target (4-hydroxyphenylpyruvate dioxygenase, HPPD) is present in non-target organisms including soil microorganisms, the targets of the three pesticides evaluated in our study are (to our best knowledge) not found in soil bacteria (IPU targets photosystem II, CHL blocks acetylcholine neurotransmission, and TCZ blocks sterol 14α-demethylase and impedes any additional modes of action on sterol biosynthesis). Thus, no direct effects of the three tested pesticides on soil bacteria were expected. Nonetheless, the transformation products of the three tested pesticides that are known for their toxicity [namely 4-isopropyl-aniline (4-IA) for IPU, TCP for CHL, and triazole transformation products for TCZ] may directly impact soil microorganisms. In addition, indirect effects of the three pesticides can be expected notably for TCZ because of close interactions between fungal and bacterial species in soil.

In any cases, the interpretation of the biological significance of the ecotoxicological effects of the tested pesticides on the composition of the soil bacterial community remains difficult in the absence of data on the normal operating range (NOR) for each studied bioindicator (such as bacterial diversity), making it challenging to define specific protection goals to protect bacterial diversity and supported soil ecosystem functions (Bell et al., 2005).

## Conclusions

Our study provides a first comprehensive lab-to-field assessment of the ecotoxocity of the three pesticides CHL, IPU, and TCZ on the soil bacterial diversity using advanced molecular methods such as PhyloChip and 16S rDNA amplicon NGS. Although the EFSA concluded that all three pesticides do not induce unacceptable changes on the soil microbial activity, one could observe that they induced minor (but significant) and transient changes in the composition of the soil bacterial community. These modern methods seem to have a good potential for the assessment of the toxicity of pesticides on the soil microbial diversity. More research is needed to define the NOR of the soil bacterial diversity in order to biologically interpret the importance of changes observed in response to pesticide exposure.

## Author contributions

VS and SN contribute to the production of the results, their exploitation and writing. CP, CC, GP, CB, PK, and EP contributed to the setup of field and lab experiments. In addition, GP and CB were in charge of soil DNA extraction. AnS and FF were in charge of pesticide residues extraction and quantification. AyS, MD-L, CM and GT were in charge of the exploitation of NGS results. DK, OS, GT and FM-L coordinated and supervised all the co-authors within the framework of the EU project LTH. They contribute actively to the preparation of this manuscript. All co-authors agreed to publish this paper to which they contribute.

### Conflict of interest statement

CB, OS, and CM were employed by ENOVEO srl. AS, GP, and FF were employed by AEIFORIA srl. The remaining authors declare that the research was conducted in the absence of any commercial or financial relationships that could be construed as a potential conflict of interest.
