# Supplementary material for: Lab to Field Assessment of the Ecotoxicological Impact of Chlorpyrifos, Isoproturon, or Tebuconazole on the Diversity and Composition of the Soil Bacterial Community
Source: Front Microbiol. 2018 Jun 29;9:1412. doi: 10.3389/fmicb.2018.01412 (PMC6034002; doi:10.3389/fmicb.2018.01412)
Supplement: Supplementary file 1 [file Data_Sheet_1.DOCX]

**Supplementary material**

**Lab to field assessment of the ecotoxicological impact of chlorpyrifos, isoproturon or tebuconazole on the diversity and composition of the soil bacterial community**

Veronika Storck^a*^, Sofia Nikolaki^b*^, Chiara Perruchon^c^, Camille Chabanis^d^, Angela Sacchi^e^, Giorgia Pertile^e^, Céline Baguelin^d^, Panagiotis A. Karas^c^, Aymé Spor^a^, Marion Devers-Lamrani^a^, Evangelia S. Papadopoulou^c^, Olivier Sibourg^d^, Cedric Malandain^d^, Marco Trevisan^f^, Federico Ferrari^e^, Dimitrios G. Karpouzas^c^, George Tsiamis^b1^, Fabrice Martin-Laurent^a1^

^a^AgroSup Dijon, INRA, Univ. Bourgogne Franche-Comté, Agroécologie, Dijon, France

^b^University of Patras, Department of Environmental and Natural Resources Management, Agrinio, Greece

^c^University of Thessaly, Department of Biochemistry and Biotechnology, Laboratory of Plant and Environmental Biotechnology, Viopolis 41500, Larissa, Greece

^d^Enoveo srl., Lyon, France

^e^Aeiforia srl, Spinoff Università Cattolica del Sacro Cuore, Fidenza, Italy

^f^Catholic University of the Sacred Heart, Department of Agronomy and Environmental and Chemistry, Piacenza, Italy

*equal first authors

^1^corresponding authors: [fabrice.martin@inra.fr](mailto:fabrice.martin@inra.fr); [gtsiamis@upatras.gr](mailto:gtsiamis@upatras.gr)


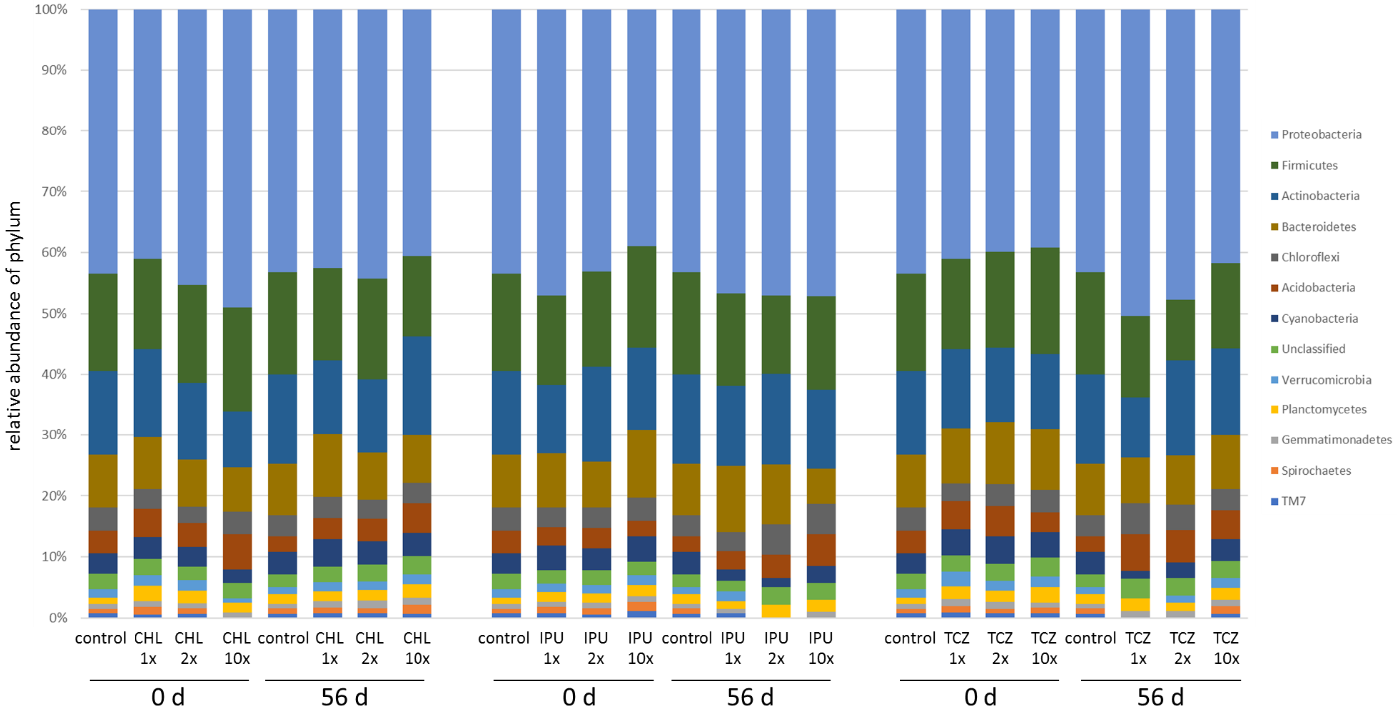


**Supplementary Figure 1**. Microcosm study – The relative abundance (%) of bacterial phyla identified by PhyloChip in the untreated (control) and pesticide-treated (CHL, IPU or TCZ at 1x, 2x, or 10x dose) soil samples at different time points (0 and 56 days after treatment). OTUs that could not be assigned to a phylum were grouped as ‘unclassified’.


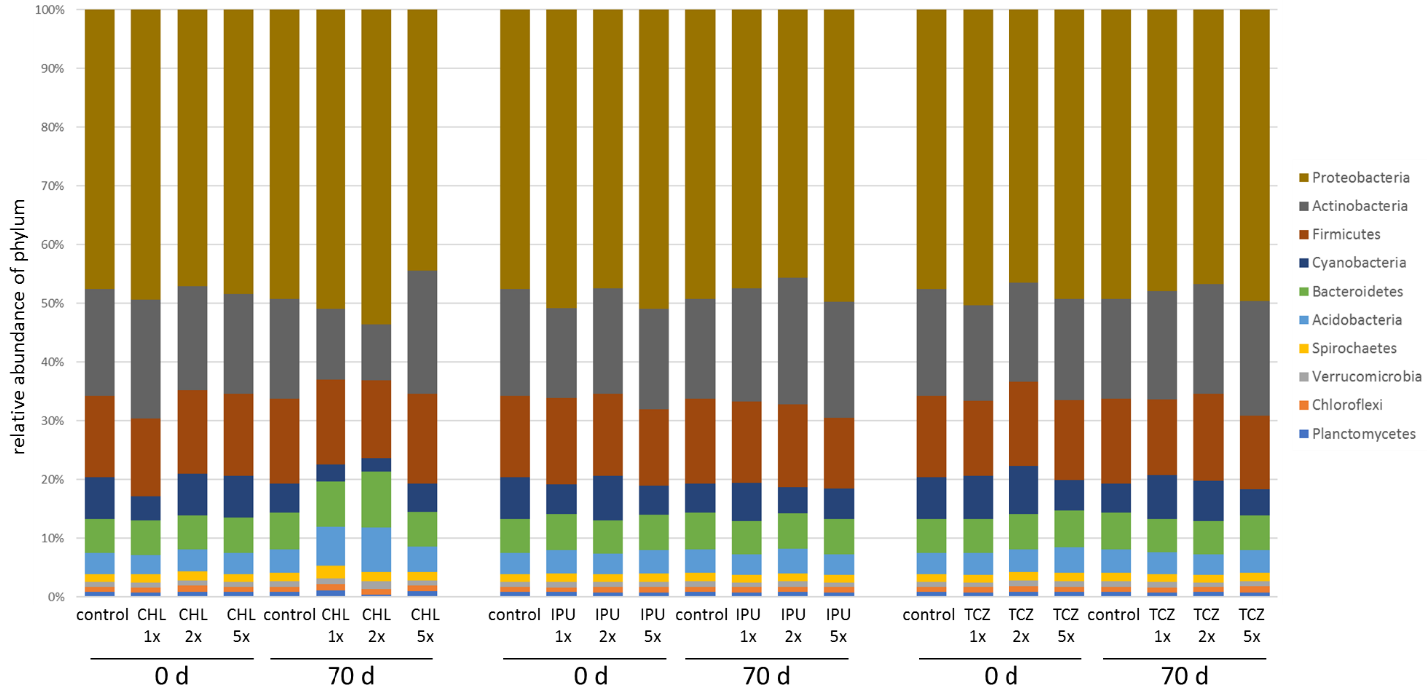


**Supplementary Figure 2.** Field study – The relative abundance (%) of bacterial phyla identified by PhyloChip in the untreated (control) and pesticide-treated (CHL, IPU or TCZ at 1x, 2x, or 10x dose) soil samples at different time points (0 and 56 days after treatment). OTUs that could not be assigned to a phylum were grouped as ‘unclassified’.


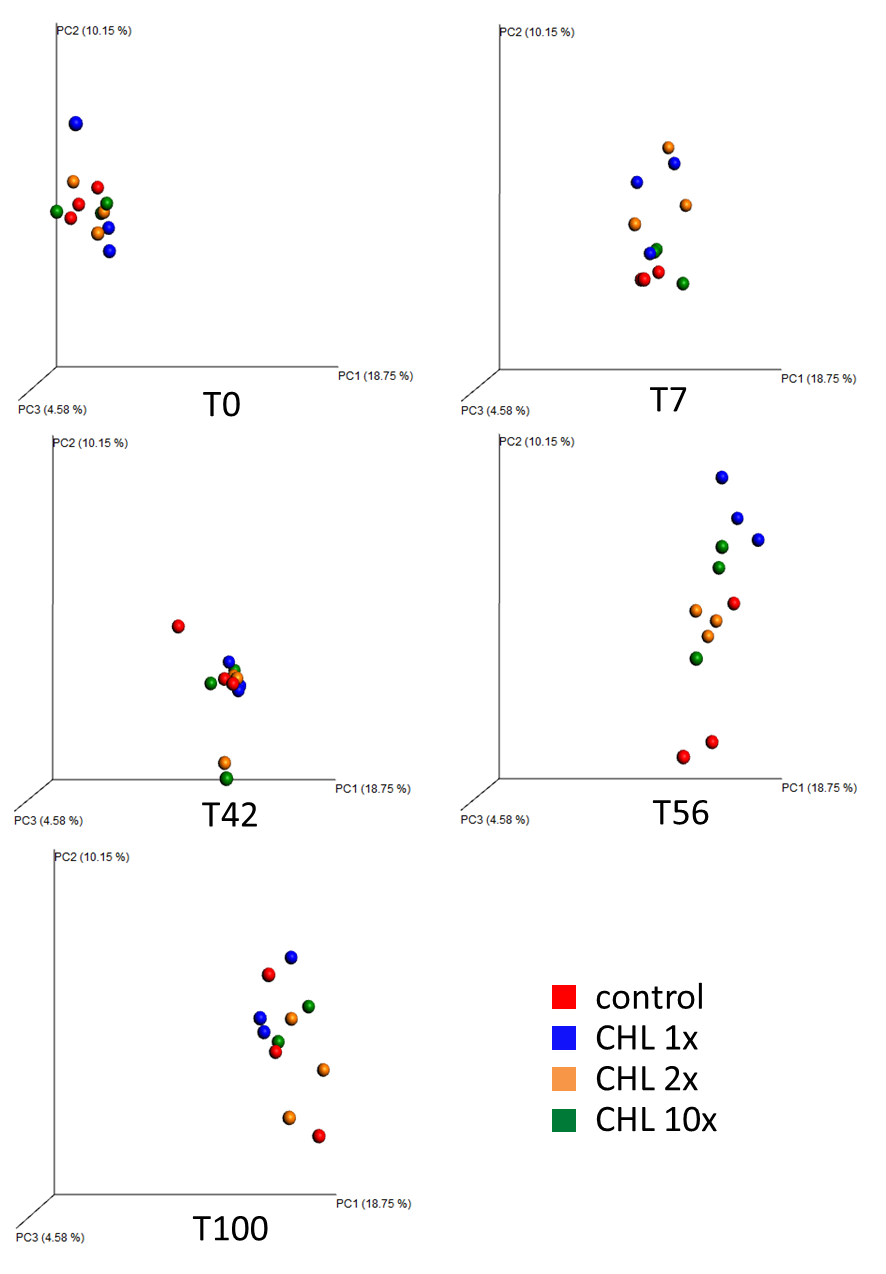


**Supplementary Figure 3.** Microcosm study - PCoA ordinations of OTU weighted unifrac distance matrices for untreated (control) and CHL-treated (1x, 2x or 10x doses) soil samples at different time points (0, 7, 42, 56 and 100 days). The percent of variance explained by each axis is given. Replicates of the same treatment are represented in same colors.


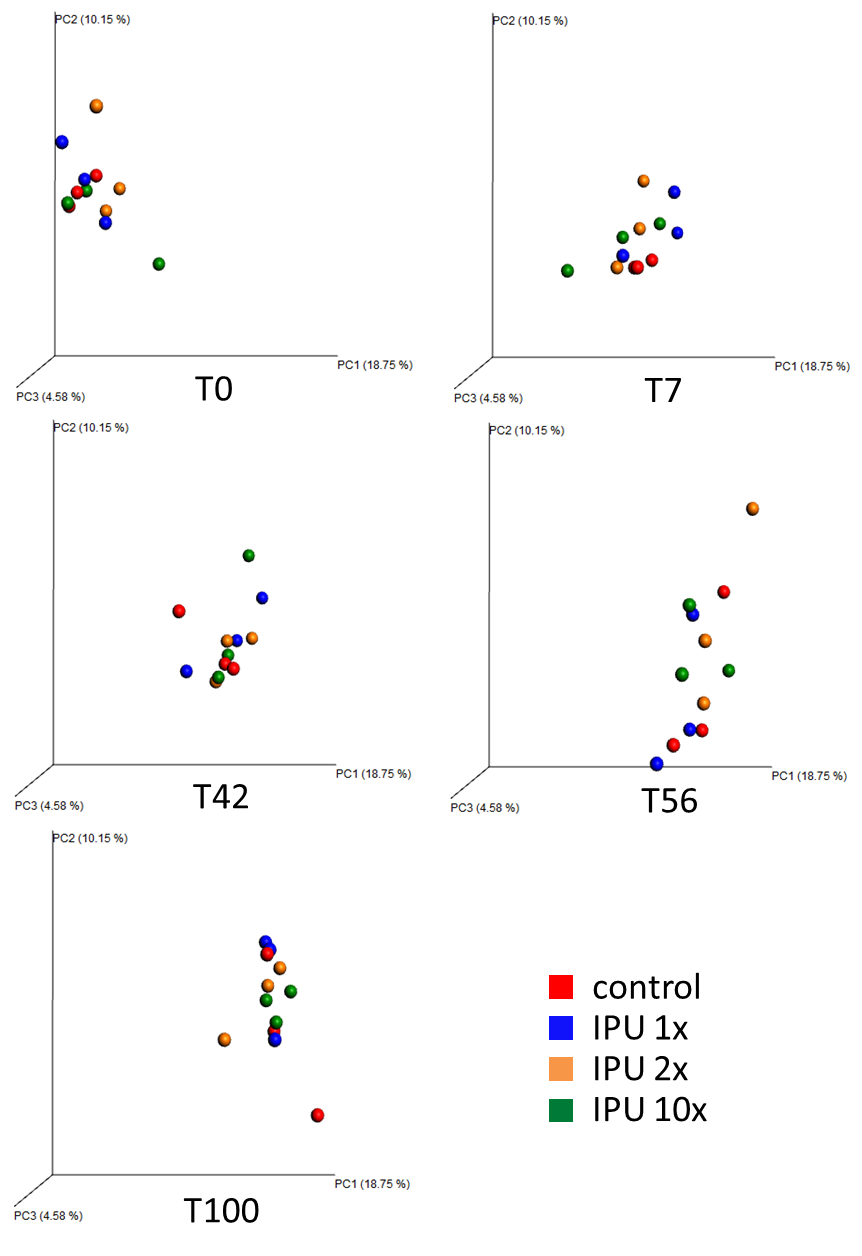


**Supplementary Figure 4**. Microcosm study - PCoA ordinations of OTU weighted unifrac distance matrices for untreated (control) and IPU-treated (1x, 2x or 10x doses) soil samples at different time points (0, 7, 42, 56 and 100 days). The percent of variance explained by each axis is given. Replicates of the same treatment are represented in same colors.


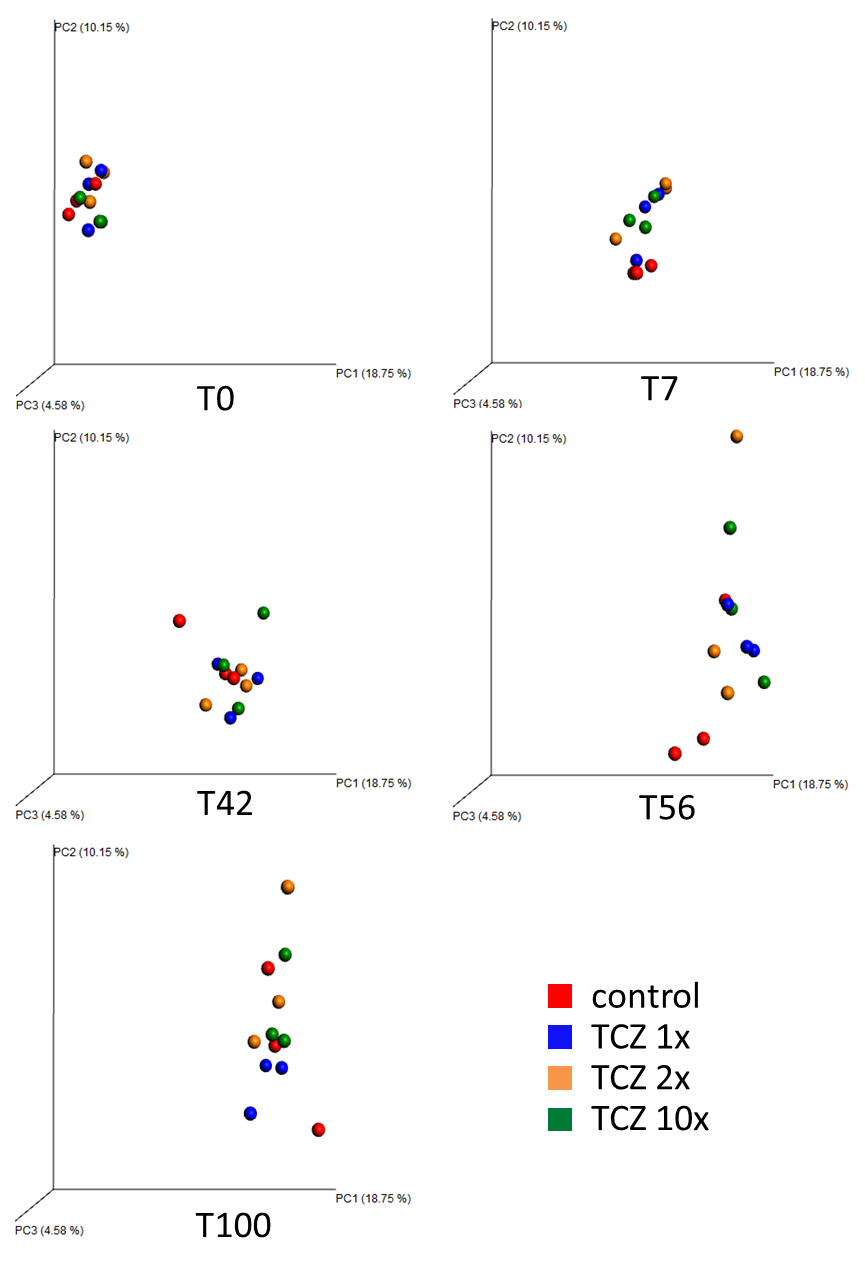


**Supplementary Figure 5.** Microcosm study - PCoA ordinations of OTU weighted unifrac distance matrices for untreated (control) and TCZ-treated (1x, 2x or 10x doses) soil samples at different time points (0, 7, 42, 56 and 100 days). The percent of variance explained by each axis is given. Replicates of the same treatment are represented in same colors.


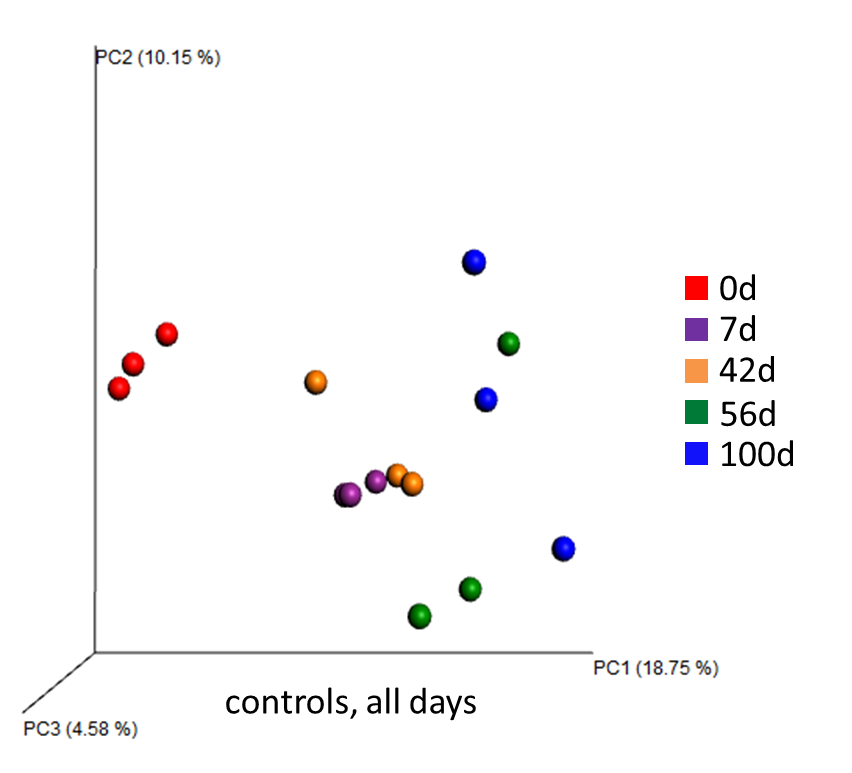


**Supplementary Figure 6.** Microcosm study - PCoA ordinations of OTU weighted unifrac distance matrices for untreated (control) soil samples at different time points (0, 7, 42, 56, 100 days). The percent of variance explained by each axis is given. Replicates of the same treatment are represented in same colors.

**
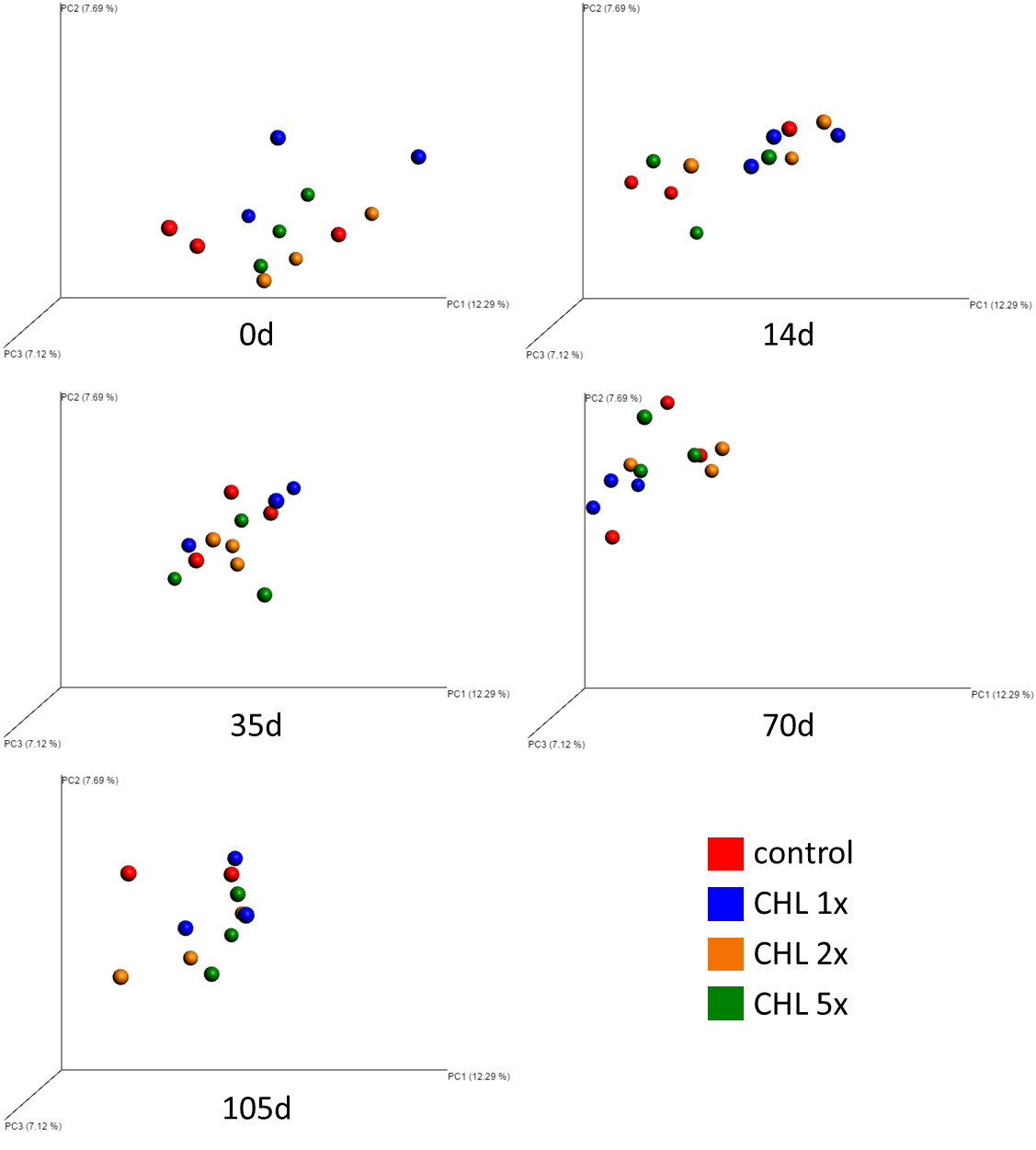
**

**Supplementary Figure 7.** Field study - PCoA ordinations of OTU weighted unifrac distance matrices for untreated (control) and CHL-treated (1x, 2x or 5x doses) soil samples at different time points (0, 14, 35, 70 and 105 days). The percent of variance explained by each axis is given. Replicates of the same treatment are represented in same colors.

**
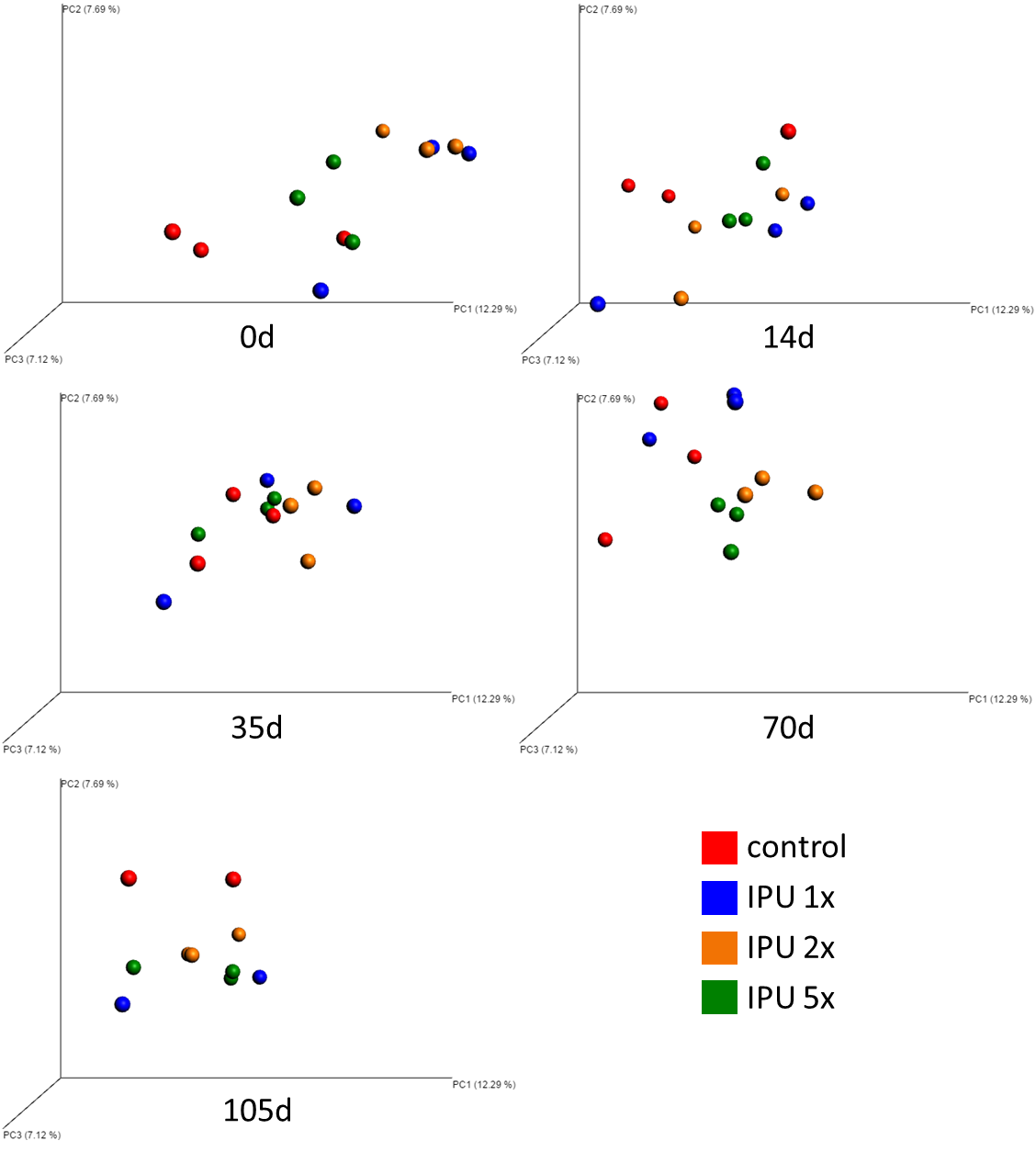
**

**Supplementary Figure 8.** Field study - PCoA ordinations of OTU weighted unifrac distance matrices for untreated (control) and IPU-treated (1x, 2x or 5x doses) soil samples at different time points (0, 14, 35, 70 and 105 days). The percent of variance explained by each axis is given. Replicates of the same treatment are represented in same colors.

**
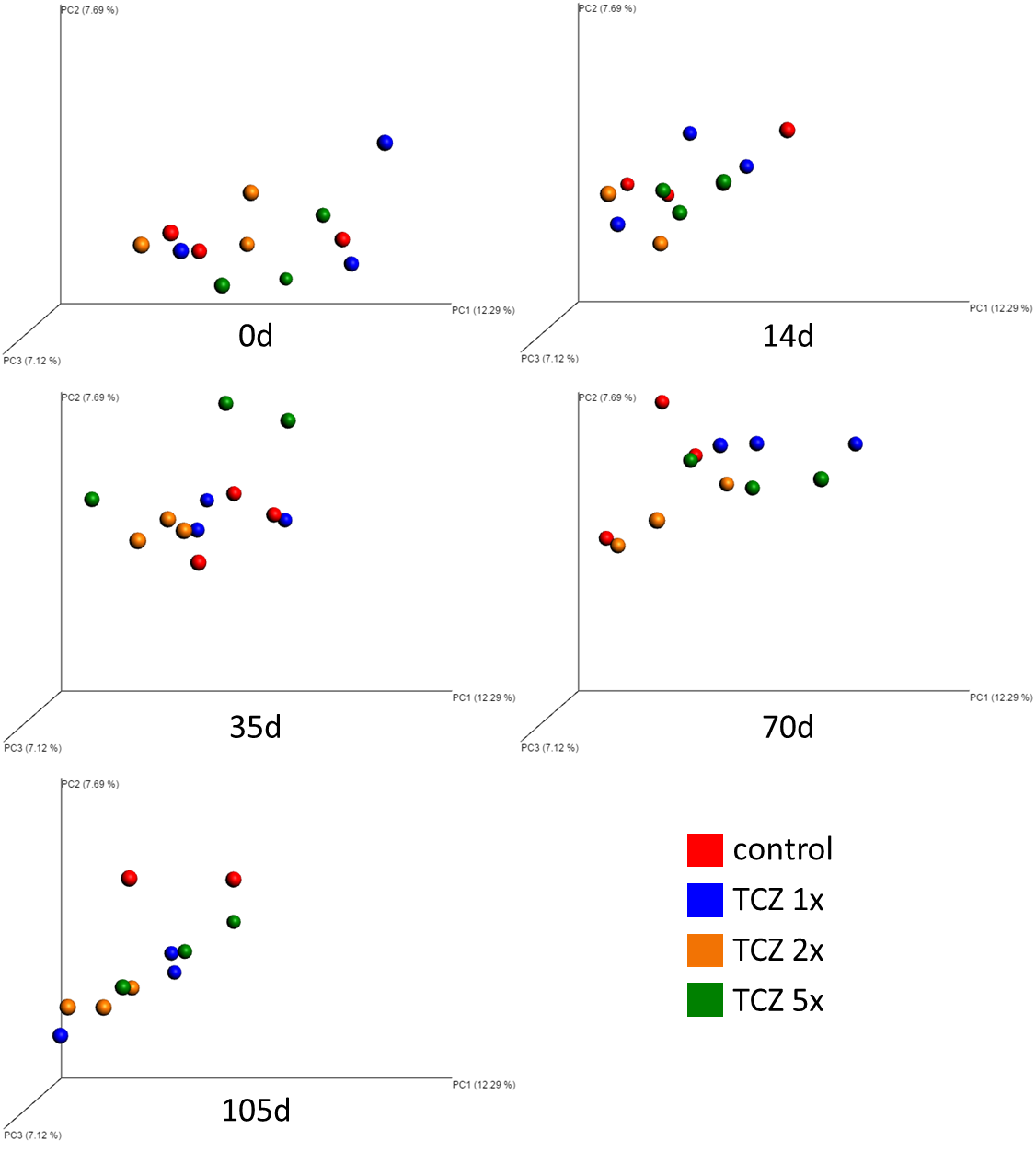
**

**Supplementary Figure 9.** Field study - PCoA ordinations of OTU weighted unifrac distance matrices for untreated (control) and TCZ-treated (1x, 2x or 5x doses) soil samples at different time points (0, 14, 35, 70 and 105 days). The percent of variance explained by each axis is given. Replicates of the same treatment are represented in same colors.

**
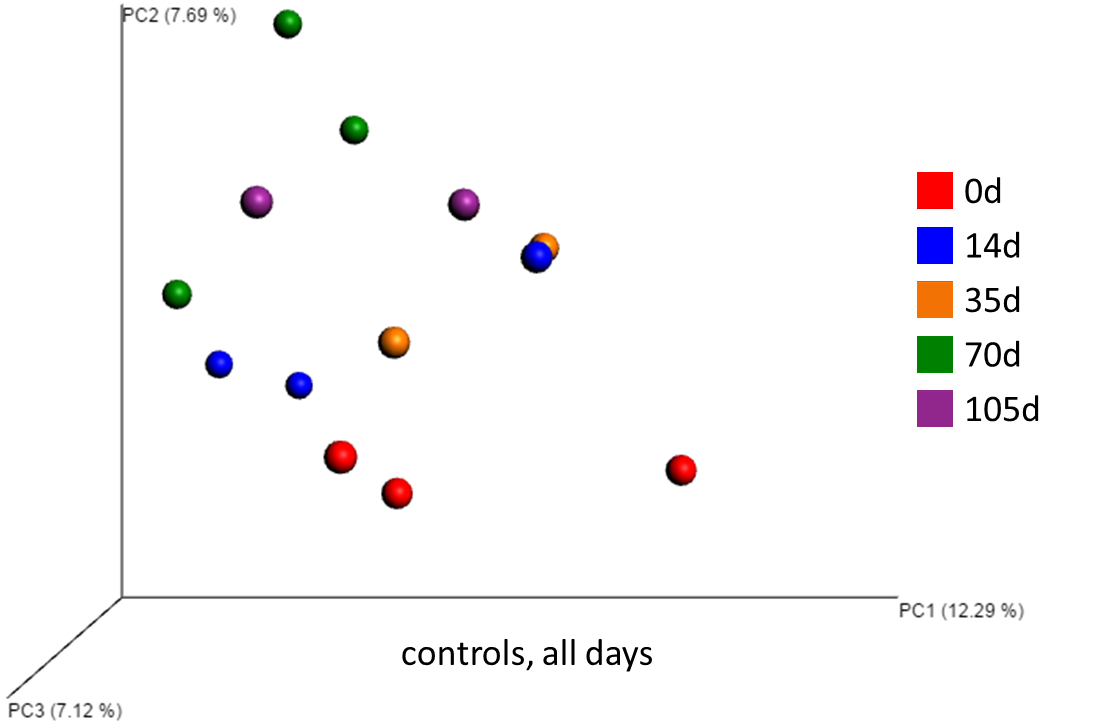
**

**Supplementary Figure 10.** Field study - PCoA ordinations of OTU weighted unifrac distance matrices for untreated (control) soil samples at different time points (0, 14, 35, 70 and 105 days). The percent of variance explained by each axis is given. Replicates of the same treatment are represented in same colors.


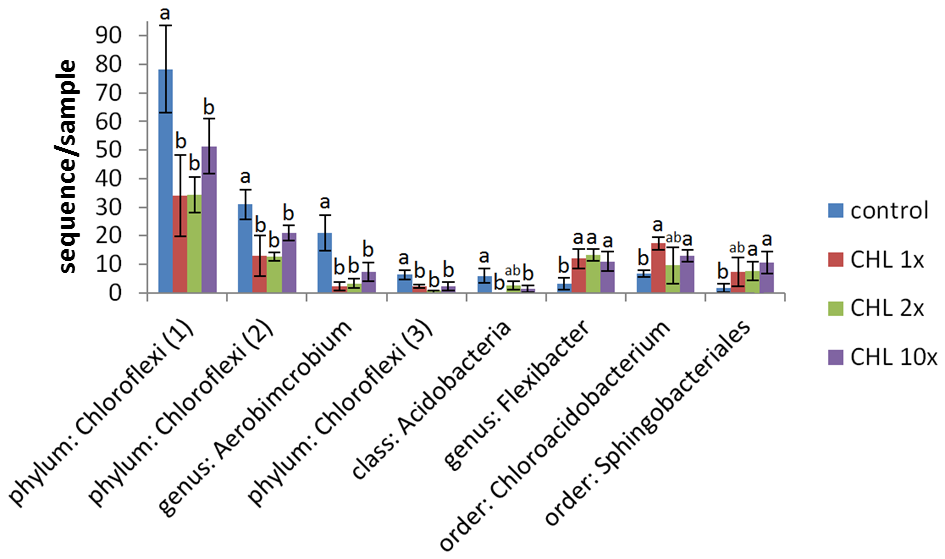


**Supplementary Figure 11.** Microcosm study - Abundance [number/sample] of OTUs responsible for significant differences of ANOSIM, detected by ‘pamR’ in untreated (control) and treated (CHL at 1x, 2x or 10x doses) soil samples at 56 days. ANOVAs were followed by the Bonferroni test per each taxon. Significant differences are indicated by letters.


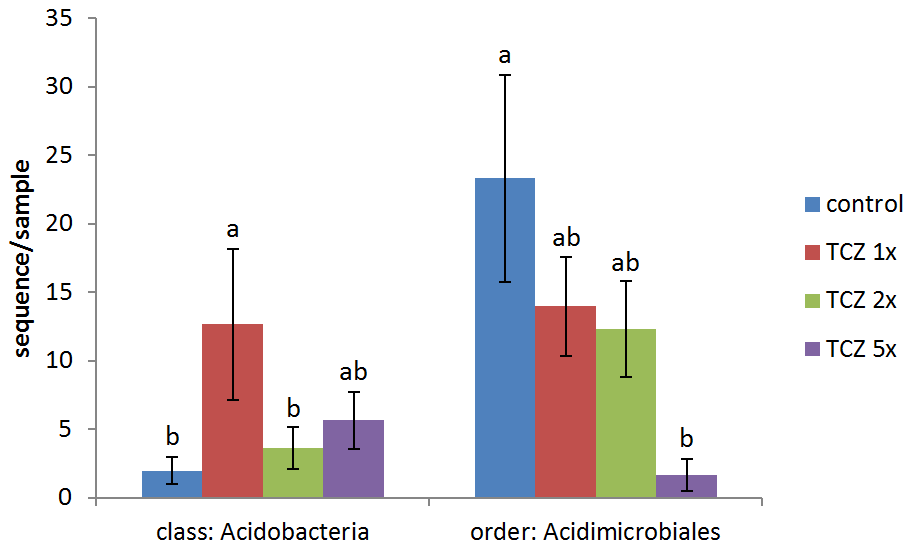


**Supplementary Figure 12.** Field study - Abundance [sequence/sample] of an OTUs responsible for significant differences of ANOSIM, detected by ‘pamR’ in untreated (control) and treated (TCZ at 1x, 2x or 10x doses) soil samples at 35 days. ANOVAs were followed by the Bonferroni test per each taxon. Significant differences are indicated by letters.
